# Molecular Dynamics of PPAR Nuclear Receptors: From Ligand Binding to Transcriptional Regulation

**DOI:** 10.3390/cells15151395

**Published:** 2026-07-31

**Authors:** Filip Stojceski, Andrea Danani

**Affiliations:** SUPSI, IDSIA—Dalle Molle Institute for Artificial Intelligence (USI-SUPSI), Polo Universitario Lugano—Campus Est, Via la Santa 1, 6962 Lugano, Switzerland; andrea.danani@supsi.ch

**Keywords:** peroxisome proliferator-activated receptors, PPARα, PPARβ/δ, PPARγ, molecular dynamics simulations, conformational dynamics, allostery, RXR heterodimer, PPAR modulators, PPAR disease-associated mutations

## Abstract

Peroxisome proliferator-activated receptors (PPARα, PPARβ/δ, and PPARγ) are ligand-regulated nuclear receptors that coordinate lipid metabolism, glucose homeostasis, inflammation, adipogenesis, differentiation, and disease-associated transcriptional programs. Although early structural models emphasized ligand-dependent stabilization of helix 12 (H12) and the activation function-2 surface (AF-2), evidence from molecular dynamics (MD) simulations, NMR, HDX-MS, crystallography, mutagenesis, and biochemical assays supports a more complex conformational-ensemble mechanism. Apo and ligand-bound PPARs populate multiple functional substates whose distributions are shifted by ligands, RXR heterodimerization, DNA binding, co-regulators, post-translational modifications, and disease-associated mutations. This review summarizes MD and integrative structural studies of PPAR conformational dynamics, isoform-specific behavior, PPAR-RXR and co-regulator interactions, ligand entry, graded activation, inverse agonism, disease-associated mutations, and phosphorylation-dependent regulation. PPARγ is the most extensively characterized isoform, whereas PPARα and particularly PPARβ/δ remain comparatively underexplored by atomistic and enhanced-sampling approaches. MD is most informative when it extends beyond post-docking pose stability and is integrated with long-timescale sampling, free-energy methods, dynamic-network analysis, and experimental validation. Future simulations should increasingly model biologically realistic assemblies containing RXR, DNA, coactivators or corepressors, disease mutations, and post-translational modifications to connect ligand chemistry with receptor allostery, transcriptional output, and disease biology.

## 1. Introduction to Peroxisome Proliferator-Activated Receptors

**Figure 1:**
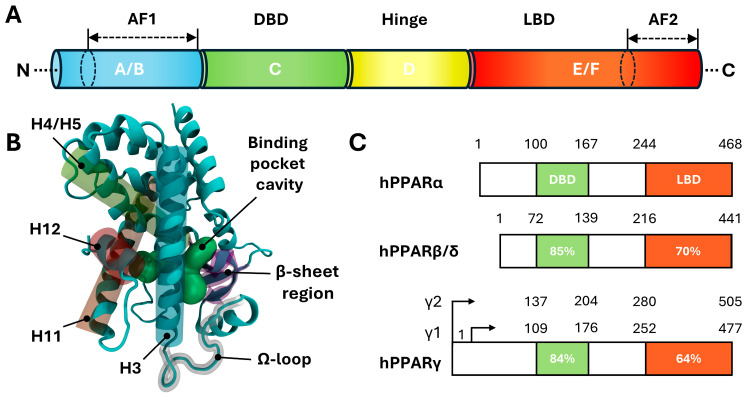
Overview of PPAR domain organization, ligand-binding-domain architecture, and isoform comparison. (**A**) Modular PPAR sequence/domain architecture, including the N-terminal A/B activation function-1 (AF-1) region, the C-domain DNA-binding domain (DBD), the D hinge region, the E/F ligand-binding domain (LBD), and the C-terminal activation function-2 (AF-2) region [9,10,11]. (**B**) Representative PPARγ LBD structure highlighting helices H3, H4/H5, H11, and H12, the Ω-loop, β-sheet region, and ligand-binding-pocket cavity (PDB ID: 3R8I [12]). (**C**) Comparison of the three PPAR isoforms (PPARα, PPARβ/δ, and PPARγ). The number inside each domain (DBD in green and LBD in red) corresponds to the percentage of amino acid sequence identity of human PPARβ/δ and PPARγ relative to PPARα [13].

Peroxisome proliferator-activated receptors (PPARs) are ligand-regulated transcription factors of the nuclear receptor superfamily that translate metabolic, nutritional, inflammatory, and pharmacological signals into gene-expression programs [1,2]. The three isoforms, PPARα, PPARβ/δ, and PPARγ, regulate partially overlapping but biologically distinct processes, including fatty-acid oxidation, lipid storage, glucose homeostasis, adipogenesis, inflammation, tissue remodeling, and cellular differentiation [3,4,5,6,7]. Like other nuclear receptors, PPARs contain a DNA-binding domain and a C-terminal ligand-binding domain (LBD), which serves as the principal structural platform for ligand recognition, heterodimerization with retinoid X receptor (RXR), and the recruitment of transcriptional co-regulators [8]. Because of these functions, PPARs occupy a central position at the interface between metabolism, chronic disease, and drug discovery. A schematic representation of the PPARs sequence domains is shown in Figure 1A.

In this context, molecular dynamics (MD) simulations have become essential for interpreting PPAR regulation at atomistic resolution. MD can connect ligand-pocket fluctuations, key structural dynamics of PPARs, and multidomain PPAR-RXR-DNA organization in ways that are not accessible from static structures alone. Here, we focus on a mechanistic reading of the MD and structural literature on PPARs, rather than attempting a systematic review or meta-analysis. Literature was selected to emphasize studies that provide mechanistic insight into PPAR conformational dynamics, ligand binding, RXR heterodimerization, co-regulator recruitment, disease-associated mutations, and post-translational regulation. Priority was given to studies combining MD with experimental structural, biochemical, biophysical, or functional evidence. Docking-based studies were included mainly when followed by MD simulations, binding-energy analysis, or experimental validation. Throughout the review, we distinguish between screening-oriented docking/short-MD workflows, which mainly test pose stability and prioritize candidates, and mechanistic MD studies, which use longer simulations, enhanced sampling, ensemble analysis, network analysis, or experimental validation to explain receptor regulation.

### 1.1. PPARs Conformational Dynamics

Early crystallographic studies of PPARγ established the canonical LBD architecture, characterized by a large Y-shaped ligand-binding pocket and an activation function-2 (AF-2) surface formed mainly by β-sheet region, helices H3, H4/H5, and H12 [14,15]. A schematic representation of the PPARs LBDs is shown in Figure 1B. For many years, PPAR activation was interpreted through a relatively simple structural model centered on helix 12 (H12). In this model, agonists stabilize H12 in an active position, completing the AF-2 coactivator-binding surface, whereas antagonists destabilize or displace H12, thereby preventing coactivator recruitment. This model was useful for interpreting ligand-bound crystal structures, but it progressively became insufficient to explain the behavior of partial agonists, inverse agonists, and selective PPAR modulators. In particular, partial agonists can activate PPARγ without fully stabilizing H12, instead stabilizing other regions of the LBD, including the β-sheet region and helix 3 [16]. Thus, the biological response cannot be explained only by the final crystallographic position of H12.

Because the PPAR LBD is flexible in solution, it is more useful to view these receptors as dynamic ensembles rather than static structures. Early biophysical studies showed that H12 is mobile and that ligand binding modulates this mobility rather than simply locking the receptor into a single active state [17]. This idea was later formalized in broader nuclear receptor dynamics models, where ligand efficacy was proposed to arise from redistribution of conformational populations within the LBD rather than from a binary inactive-to-active transition [18]. In this framework, apo PPARs should not be treated simply as passive inactive structures. They are dynamic ensembles that already sample conformations relevant to activation, repression, ligand entry, and co-regulator binding.

MD contributed strongly to this shift by providing atomistic access to protein motions that are invisible in single crystal structures. In PPARγ, enhanced-sampling simulations showed that the apo receptor can access multiple H12 orientations, including antagonist-like conformations, indicating that inactive and active-like states are part of the intrinsic conformational landscape [19]. Other computational studies focused specifically on H12 conformational diversity further supported the idea that ligand-free PPARγ samples multiple H12 substates rather than a single inactive arrangement [20]. Together, these observations make conformational selection a plausible framework for PPARγ, because ligands appear to stabilize substates that are already sampled by the apo receptor.

The strongest evidence for this ensemble-based mechanism came from integrative studies performed by Chrisman et al., using MD simulations, fluorine-19 NMR, HDX-MS, crystallography, and co-regulator recruitment assays to define the conformational ensemble of the PPARγ AF-2 surface [21]. They showed that apo PPARγ and partial-agonist-bound PPARγ populate broad ensembles with multiple accessible conformations, whereas full agonists and inverse agonists restrict the ensemble toward fewer conformations compatible with coactivator or corepressor binding, respectively. A key contribution of Chrisman et al. was to connect ligand efficacy with AF-2 population redistribution, rather than with a single crystallographic LBD conformation.

Apo and repressed PPARγ states were later shown to be even more heterogeneous than previously appreciated. Heidari and co-workers combined NMR, accelerated MD, HDX-MS, mutagenesis, and crystallography to define multiple structurally distinct repressive states of PPARγ [22]. Their work revealed that the H3 charge-clamp region is highly variable in apo PPARγ and becomes stabilized by efficacious ligands. They also identified an Ω-loop to helix transition linked to inverse agonism and disruption of a tripartite salt-bridge network. This shifted the mechanistic interpretation beyond H12 alone and placed H3, the Ω-loop, and the AF-2 surface within the same activation-repression network.

Recent simulations have provided a more quantitative description of this landscape. Falbo and colleagues combined classical MD, Gaussian accelerated MD, machine-learning (ML) clustering, and well-tempered metadynamics to reconstruct transitions between apo, agonist-like, partial agonist-like, and antagonist-like PPARγ-LBD conformations [23]. In that model, apo PPARγ is better described as a metastable ensemble stabilized by transient intramolecular contacts, rather than as a fully disordered receptor. Consistent with this direction, Zhao et al. recently combined extensive MD simulations, Markov-state modeling, and energetic analysis to map the allosteric landscape of PPARγ, further supporting the view that receptor function emerges from transitions among ligand-selectable conformational states [24].

However, PPAR conformational dynamics are not restricted to local motions of the ligand-binding pocket. MD studies of PPARα demonstrated that ligand binding reorganizes collective motions, hydrogen-bond networks, correlated residue fluctuations, and intramolecular communication pathways throughout the LBD [25]. Using apo, agonist-bound, and antagonist-bound PPARα systems, Basith et al. showed that activation involves long-range signal propagation from the orthosteric pocket to distal regulatory surfaces, including the AF-2 region. Their results indicated that different ligand states reshape the internal communication architecture of the receptor. Thus, the PPARα data argue for an allosteric interpretation in which ligand binding reorganizes motions across the LBD, not only around the orthosteric pocket.

PPAR-RXR complexes provide another important layer of evidence for allosteric regulation. PPARs function physiologically as heterodimers with RXR, and structural studies of PPARγ-RXRα complexes revealed an asymmetric multidomain architecture [26]. NMR, crystallography, and HDX-MS studies further showed that RXR and permissive partners such as PPARγ communicate allosterically through the heterodimer interface [27]. MD and statistical network analyses of the PPARγ-RXRα complex also identified correlated motions and preferential allosteric pathways linking Ω-loops, DNA-binding domains, and ligand-binding regions [28]. Taken together, the heterodimer studies make isolated LBD simulations an incomplete model for transcriptional regulation, because RXR, DNA, and co-regulators reshape the same dynamic networks.

The field has therefore moved away from a simple H12-switch model toward an ensemble-based view of PPAR regulation. In this model, apo PPARs sample multiple substates that differ in H12 orientation, AF-2 organization, Ω-loop structure, H3 charge-clamp geometry, and long-range communication pathways. Ligands, RXR heterodimerization, DNA binding, post-translational modifications, and co-regulators modulate receptor function by reshaping this pre-existing ensemble. MD simulations, especially when integrated with NMR, HDX-MS, crystallography, and biochemical assays, are a powerful tool for understanding PPARs activation, repression, partial agonism, inverse agonism, and selective modulation.

### 1.2. Comparison Among PPARα, PPARβ/δ, and PPARγ

Although PPARα, PPARβ/δ, and PPARγ share the same nuclear receptor fold and a broadly conserved LBD, their conformational dynamics are not equivalent (Figure 1C). The three receptors possess homologous Y-shaped ligand-binding pockets, but subtle differences in pocket volume, polarity, residue composition, and helix flexibility influence ligand selectivity, activation strength, and allosteric communication [29,30,31,32]. These structural differences are especially important for MD studies because small variations in the ligand-binding cavity can produce different patterns of H12 stabilization, AF-2 surface organization, Ω-loop mobility, and intramolecular signal propagation.

PPARγ is by far the best-characterized isoform from the perspective of conformational dynamics. This predominance reflects its pharmacological importance in several diseases, including diabetes, obesity, inflammation, and cancer. PPARγ has become the reference system for understanding how ligand efficacy emerges from redistribution of conformational ensembles rather than from a simple static active/inactive transition. MD, NMR, HDX-MS, crystallography, and enhanced-sampling simulations have shown that apo PPARγ samples multiple substates involving H12, the AF-2 surface, the Ω-loop, and the H3 charge-clamp region [21,22,23]. Full agonists generally restrict this ensemble toward coactivator-compatible conformations, whereas partial agonists and repressive ligands stabilize alternative regions or repressive states [16,21,22,23,33,34,35].

A critical feature of PPARγ is the extent to which ligands can exploit multiple regions of the LBD. Classical full agonists, such as thiazolidinediones, typically stabilize H12 through interactions with the polar arm of the binding pocket, thereby favoring AF-2 formation. In contrast, many partial agonists preferentially stabilize the β-sheet region, H3, or alternative pocket regions without fully locking H12 in the canonical active orientation [16,34]. Structural and dynamic studies have therefore established PPARγ as a highly plastic receptor in which ligand efficacy depends on which elements of the LBD are stabilized and which dynamic pathways remain accessible. This plasticity also explains why ligands with similar crystallographic poses can produce different transcriptional responses.

Recent studies have further shown that PPARγ ligand recognition itself is dynamic. Cooperative co-binding experiments demonstrated that synthetic ligands can displace endogenous fatty acids from the orthosteric pocket toward an alternate site near the Ω-loop, highlighting the ability of PPARγ to accommodate multiple ligands and redistribute conformational strain across the LBD [35]. A recent MD/HINT-based analysis of the PPARγ-rosiglitazone system further emphasized the dynamic role of the Ω-loop in ligand-mediated activation [36]. This study showed that oleic-acid co-binding can stabilize intramolecular interaction patterns between the flexible Ω-loop and helix H3, thereby providing an energetic interpretation of cooperative ligand effects on receptor flexibility [36]. In addition, structural and computational work suggested that PPARγ agonists may bind through a two-step mechanism involving an initial encounter complex followed by transition into the orthosteric binding pose [37]. PPARγ ligand recognition therefore cannot be reduced to final ligand pose stability: ligand entry, transient binding states, pocket remodeling, and distal co-regulator surfaces all contribute to the functional outcome.

Compared with PPARγ, the intrinsic conformational dynamics of PPARα have been less extensively explored. PPARα is primarily associated with fatty acid oxidation, lipid catabolism, and fibrate pharmacology [1,3,7]. Early structural studies revealed conserved activation mechanisms between PPARα and PPARγ, particularly in agonist stabilization of H12 and the AF-2 surface [29,30]. However, fewer studies have focused specifically on apo or antagonist-bound PPARα. The most direct MD analysis remains the work comparing apo, agonist-bound, and antagonist-bound PPARα, which showed that ligand state strongly influences collective motions, hydrogen-bond networks, dynamic cross-correlations, and communication pathways across the LBD [25]. This PPARα study is important because it indicates that the allosteric principles identified in PPARγ are not unique to that isoform. In PPARα, ligand binding reorganizes intramolecular communication from the orthosteric pocket toward distal regulatory regions, including the AF-2 surface [25]. However, PPARα lags behind PPARγ in integrative characterization, especially regarding studies that combine long-timescale MD, enhanced sampling, NMR, HDX-MS, and co-regulator assays.

The situation is even more limited for PPARβ/δ, despite its roles in fatty acid metabolism, skeletal muscle physiology, inflammation, wound healing, and cancer biology [38]. Structurally, PPARβ/δ shares the general PPAR LBD architecture, but its pocket properties differ in ways that influence ligand selectivity [31,32]. A key early observation was that recombinant human PPARβ/δ LBD can be locked in an activated conformation by endogenous fatty acids, suggesting that this isoform may have a strong tendency to adopt active-like states under certain ligand-bound conditions [31]. This feature complicates direct comparison with apo PPARγ, where multiple inactive, active-like, and repressive states have been characterized in detail.

Structural studies of PPARβ/δ-selective ligands have identified specific residues and pocket features that contribute to subtype selectivity. For example, the crystal structure of PPARβ/δ bound to GW0742, together with comparative and computational analyses, suggested that residues such as V312 and I328 contribute to selective ligand accommodation and help distinguish the PPARβ/δ pocket from those of PPARα and PPARγ [32]. These data suggest that residue-level differences in pocket shape and steric constraints are expected to influence ligand residence, pocket breathing, H12 stabilization, and communication with AF-2. However, compared with PPARγ, relatively few studies have explicitly used long-timescale or enhanced-sampling MD to map the apo conformational ensemble of PPARβ/δ.

Pan-PPAR and dual-PPAR ligand studies provide useful opportunities to compare isoform dynamics directly. Structural studies of pan-agonists and dual agonists have shown that related ligands can adopt subtly different binding modes across PPARα, PPARβ/δ, and PPARγ, reflecting both conserved activation motifs and isoform-specific pocket constraints [30,34]. In an MD-based comparative study of the pan-agonist chiglitazar, Sullivan and co-workers docked the ligand into experimentally resolved structures of human PPARα, PPARβ/δ, and PPARγ, followed by 3 μs simulations for each receptor–ligand complex [39]. In those simulations, chiglitazar engaged all three isoforms, but with differences in residue-level interactions and binding energetics. This indicates that pan-agonism depends on both conserved AF-2 stabilization and isoform-specific pocket accommodation [39].

Based on the current literature, PPARγ is still the dynamic benchmark of the family, PPARα as a mechanistically related but underexplored system, and PPARβ/δ as the least developed from the perspective of apo and enhanced-sampling MD. PPARγ studies have already established a mature ensemble model involving H12, AF-2, H3, β-sheet, Ω-loop, ligand entry, repressive states, and co-regulator interactions [21,22,23,33,35,37]. PPARα studies support a similar allosteric framework but remain fewer and less experimentally integrated [25,29,30]. PPARβ/δ structural work has clarified selectivity determinants and active-state stabilization, but its intrinsic apo dynamics remain insufficiently mapped [31,32]. Therefore, a major conclusion from the isoform comparison is that the mechanistic principles derived from PPARγ should not be automatically generalized to the entire PPAR family without caution. The three isoforms share a conserved nuclear receptor architecture, but their dynamic landscapes are shaped by different ligand-pocket geometries, tissue-specific ligands, activation tendencies, and co-regulator preferences. A summary of key MD-oriented studies supporting conformational dynamics of PPAR nuclear receptors is shown in Table S1—Supplementary Materials (SM).

## 2. PPAR-Protein Interactions: RXR Heterodimers, Co-Regulators, Kinases, and Transcriptional Complexes

**Figure 2:**
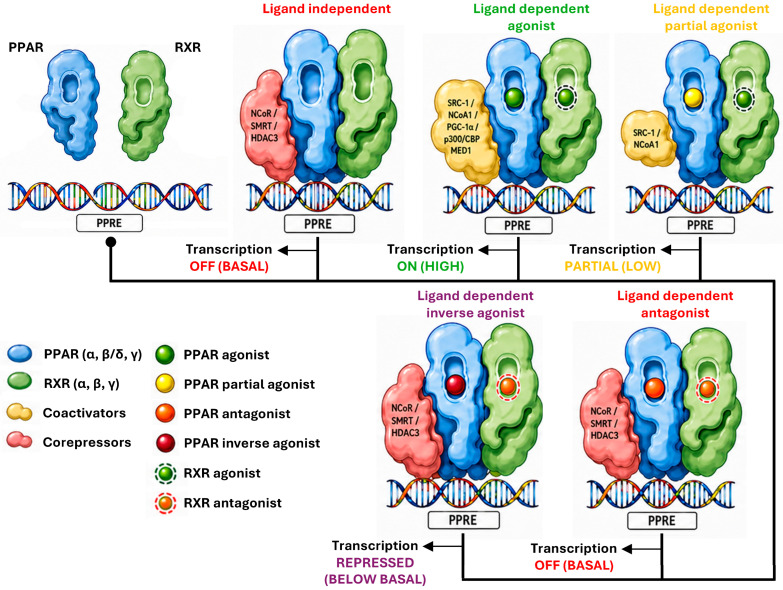
Schematic representation of PPAR-RXR heterodimer-mediated transcriptional regulation at PPAR response elements (PPREs) [40,41]. PPARs function predominantly as heterodimers with RXR and regulate target-gene transcription through ligand-dependent changes in coactivator and corepressor recruitment. In the ligand-independent state, the DNA-bound PPAR-RXR complex is depicted as associated with corepressors (e.g., NCoR, SMRT, and HDAC3) maintaining low basal transcriptional activity. Binding of a full agonist to the PPAR ligand-binding domain stabilizes a coactivator-compatible receptor state and promotes recruitment of coactivators (e.g., SRC-1/NCoA1, PGC-1α, p300/CBP, and MED1) resulting in a high transcriptional activation. Partial agonists produce lower and potentially selective coactivator recruitment, consistent with stabilization of a smaller population of fully active AF-2 conformations and, consequently, graded transcriptional activation. Neutral antagonists block agonist-dependent activation and favor corepressor retention or re-recruitment but do not necessarily reduce receptor activity below its basal level. In contrast, inverse agonists stabilize repressive, corepressor-compatible conformational states and decrease constitutive transcriptional activity below the basal level. RXR agonist binding may further enhance transcriptional activation in permissive PPAR-RXR heterodimers [42], whereas RXR antagonist occupancy may oppose RXR-dependent activation. Solid ligand symbols indicate PPAR ligands, while dashed ligand symbols indicate optional RXR agonist or antagonist occupancy.

PPARs do not function as isolated ligand-binding domains. In cells, they act as multidomain transcription factors that heterodimerize with RXRs, bind PPAR response elements (PPREs) on DNA, recruit coactivators or corepressors, and undergo post-translational regulation by kinases. A schematic representation of PPAR-RXR heterodimer-mediated transcriptional regulation is shown in Figure 2. Consequently, MD studies of PPARs have progressively moved from isolated LBD toward more complex assemblies that include RXR, DNA, co-regulator peptides, phosphorylation sites, and kinase recognition interfaces. This shift is crucial because ligand binding, co-regulator recruitment, DNA recognition, and phosphorylation are not independent events. Instead, they are connected through long-range allosteric communication across the receptor complex. A summary of MD-focused and integrative studies on PPAR–protein interactions is shown in Tables S2—SM.

### 2.1. PPAR-RXR Heterodimers as Dynamic Allosteric Assemblies

PPAR transcriptional activity depends strongly on heterodimerization with RXR. The PPARγ-RXRα structure bound to DNA, ligands, and coactivator peptides revealed that the complex is highly asymmetric, with the PPARγ LBD contacting multiple domains of the heterodimer [26]. This structure provided the first clear structural basis for the idea that ligand binding to one domain can influence DNA binding, co-regulator recruitment, and interdomain communication elsewhere in the complex.

Later MD studies showed that this coupling is also reflected in the motions of the complex. Venäläinen et al. investigated the molecular mechanism of allosteric communication in the human PPARα-RXRα heterodimer [43]. Venäläinen et al. helped establish the view that PPAR-RXR heterodimers should be considered allosteric signaling units rather than independent ligand-binding modules. In this framework, ligand binding to PPARα or RXRα reshapes interdomain contacts and modifies the dynamic communication between the two partners. Additional all-atom MD simulations of the PPAR-RXR complex showed that regulatory molecules can reshape the relative orientation and internal contacts of the heterodimer, supporting the view that ligand binding, receptor-receptor communication, and transcriptional regulation are dynamically coupled rather than independent structural events [44].

A more detailed atomistic description of heterodimer-level communication was provided by Ricci et al., who used MD simulations and statistical network analysis to investigate allosteric pathways in the PPARγ-RXRα complex [28]. Their simulations revealed interdependent motions involving the Ω-loops, the PPARγ DNA-binding domain, and interdomain contacts sensitive to conformational changes and mutations. Correlated-motion analysis also identified preferential allosteric routes with convergence centers composed largely of polar residues. The Ricci et al. analysis links transcriptional regulation to coordinated motions between ligand-binding, DNA-binding, and dimerization interfaces, making a purely local ligand-induced mechanism unlikely [28].

The functional importance of DNA in this allosteric network was further highlighted by studies showing that DNA binding can propagate conformational effects into the receptor complex. Indeed, de Vera et al. demonstrated that DNA response elements can regulate co-regulator nuclear receptor interactions by altering the thermodynamics of PPARγ-RXRα ligand and co-regulator binding [45]. Although this work was not primarily an MD study, it is crucial for interpreting simulations because it shows that DNA is not a passive scaffold. Instead, DNA sequence and receptor binding can reshape the conformational state of the heterodimer and influence the affinity of ligands and co-regulators. More recently, microsecond-scale MD simulations were used to compare RXRα-PPARγ heterodimers bound or unbound to DNA in the presence of co-crystallized ligands [46]. These simulations directly addressed how DNA binding modifies the structural and energetic behavior of the heterodimer. This comparison makes clear why isolated LBD models are limited. In detail, DNA binding, dimerization, and ligand occupancy jointly define the dynamic architecture of the PPARγ-RXRα transcriptional complex.

### 2.2. Coactivator Recruitment and AF-2 Dynamics

Coactivator recruitment is usually linked with stabilization of the AF-2 surface, particularly H12, which contributes to the binding groove for LXXLL-containing coactivator motifs. Early crystallographic work showed how agonist-bound PPARγ assembles a coactivator-compatible AF-2 surface [14,26]. However, the coactivator binding is not simply a consequence of one rigid H12 position, but it depends on the population of AF-2 conformations sampled by the receptor. Chrisman et al. demonstrated that different ligands reshape the PPARγ AF-2 conformational ensemble and thereby influence co-regulator preference [21]. Full agonists restrict the ensemble toward coactivator-compatible states, whereas partial agonists preserve broader conformational heterogeneity. For protein–protein interactions, this means that coactivator recruitment is governed by ensemble selection, in which coactivators bind more efficiently when the ligand has increased the population of compatible AF-2 conformations.

The importance of coactivator-linked allostery also appears in full-complex simulations. Lemkul and co-workers performed extensive MD simulations of the PPARγ-RXRα-DNA complex to examine how phosphorylation, ligands, and coactivator peptides influence collective motions [47]. Their simulations showed that perturbations at the PPARγ phosphorylation site can affect the LBD-LBD dimerization interface and the AF-2 coactivator-binding region. The study therefore provided atomistic evidence that coactivator recruitment is sensitive not only to local AF-2 structure but also to long-range motions across the full receptor complex. These observations are consistent with the broader view that ligand binding, coactivator recognition, and interdomain dynamics are coupled processes.

### 2.3. Corepressor Binding and Repressive PPARγ Conformations

Corepressor recruitment is a less classical, but highly relevant, mode of PPAR regulation. Whereas coactivator binding is generally associated with the canonical active AF-2 conformation, ligand-induced corepression can involve structurally diverse arrangements. This has been especially well studied for PPARγ inverse agonists and antagonists.

Repressive PPARγ conformations are best understood as an ensemble of ligand-, peptide-, and H12-dependent states rather than as a single inactive structure. Heidari et al. combined NMR, accelerated MD, HDX-MS, mutagenesis, and crystallography to define multiple repressive PPARγ states, including conformations in which the H3/H4 charge-clamp region stabilizes corepressor peptide binding [22]. Structural work by Shang et al. further showed that inverse agonist/corepressor-bound PPARγ can adopt a repressive architecture in which H12 is displaced from the canonical active position and occupies the orthosteric pocket [33]. Importantly, PRE-NMR and crosslinking MS supported the presence of this state in solution, making it a useful experimental reference for MD simulations of repression.

Peptide-dependent experiments further indicate that repression is not controlled only by ligand identity. Zheng et al. showed that ligands and regulatory peptides reshape the H12 conformational landscape [48], while Frkic et al. demonstrated that corepressor binding can also stabilize alternate ligand conformations and expand H12 ensembles [49]. Together, these studies suggest a bidirectional mechanism: ligands influence corepressor recruitment, but corepressor binding can also remodel ligand pose and receptor dynamics. This suggests that future MD studies of antagonists or inverse agonists should include corepressor peptides whenever the biological question involves repression, rather than simple ligand-pocket stability.

### 2.4. Kinase Recognition and Phosphorylation-Dependent Dynamics

PPARγ regulation by phosphorylation adds another layer of complexity in the protein–protein interaction system. Cdk5-mediated phosphorylation of PPARγ at S273 in PPARγ2, corresponding to S245 in PPARγ1, is associated with obesity-linked dysregulation of insulin-sensitizing gene programs [50]. This regulatory event is especially interesting for MD because the phosphorylation site lies in a region that can communicate with RXR, DNA-binding domains, and the AF-2 coactivator-binding surface.

Mottin et al. investigated the molecular recognition of PPARγ by the Cdk5/p25 kinase complex using protein-protein docking and adaptive biasing force simulations [51]. Their work directly addressed how Cdk5/p25 may recognize and access the PPARγ phosphorylation site. In this model, β-sheet and Ω-loop conformations influence Cdk5 accessibility, linking ligand-induced stabilization of this region to reduced phosphorylation. This provided a mechanistic bridge between ligand binding, receptor dynamics, and kinase recognition.

Lemkul et al. extended this problem to the full PPARγ-RXRα-DNA complex [47]. Their MD simulations showed that phosphorylation alters collective motions throughout the receptor assembly, affecting the LBD-LBD dimerization interface and the AF-2 coactivator-binding region. Notably, phosphorylation did not act only locally at S245. Instead, it changed the dynamic coupling between PPARγ, RXRα, and DNA, supporting a model in which phosphorylation modifies transcriptional output through long-range allosteric effects.

Montanari and colleagues later combined in silico and solution-based approaches to investigate PPARγ phosphorylation and its inhibition mechanism [52]. This supports the idea that ligands can inhibit Cdk5-mediated phosphorylation through direct and allosteric mechanisms involving stabilization of structural regions around the phosphorylation site. From an MD perspective, the implication is that ligand efficacy should not be evaluated only through transcriptional agonism. Ligands can also produce therapeutic effects by modifying protein-protein recognition surfaces involved in kinase access and post-translational regulation.

### 2.5. Emerging View: PPAR–Protein Interactions as Coupled Dynamic Networks

The available MD and structural studies show that PPAR–protein interactions are governed by coupled dynamic networks. RXR heterodimerization links PPAR LBD to another ligand-responsive receptor and creates interdomain communication routes. DNA binding modifies the conformational and thermodynamic behavior of the heterodimer. Coactivator and corepressor peptides select and reshape AF-2/H12 ensembles. Kinase recognition depends on the accessibility and stabilization of dynamic regions such as the β-sheet and Ω-loop. These processes are interconnected rather than independent.

For future MD work, this implies that simulations of isolated LBDs are valuable for dissecting ligand-pocket and H12 behavior, but they cannot fully capture transcriptional regulation. More realistic models should include RXR, DNA response elements, coactivator or corepressor peptides, phosphorylation states, and, where relevant, kinase complexes. Enhanced sampling and network-based analyses will be particularly useful for identifying the pathways through which local perturbations—ligand binding, phosphorylation, or peptide recruitment—propagate across multidomain PPAR assemblies.

The current literature is still strongly biased toward PPARγ. PPARα-RXR allostery has been explored computationally, but less extensively than PPARγ-RXRα. In a similar way, PPARβ/δ protein-interaction dynamics remain comparatively underdeveloped. A useful next step would be to compare PPARα, PPARβ/δ, and PPARγ in matched heterodimeric and DNA-bound assemblies. Such studies would clarify whether the dynamic principles established for PPARγ apply across the family or whether each isoform uses distinct protein-interaction networks to regulate transcription.

## 3. PPAR Disease-Associated Mutations: Structural and Molecular Dynamics Perspectives

Disease-associated mutations in PPARs provide a powerful opportunity to connect receptor structure, conformational dynamics, transcriptional regulation, and pathology. Among the three PPAR isoforms, disease-linked mutations have been most extensively characterized for PPARγ, particularly in familial partial lipodystrophy type 3 (FPLD3), severe insulin resistance, type 2 diabetes, dyslipidemia, and obesity-associated metabolic dysfunction [53,54,55,56,57]. In contrast, PPARα and PPARβ/δ genetic variants are more often studied through population genetics and association studies than through atomistic MD. As a result, most mutation-focused MD studies are still centered around PPARγ, while comparable mechanistic simulations for PPARα and PPARβ/δ remain limited.

PPARγ is the master regulator of adipocyte differentiation and adipose tissue function, and loss-of-function PPARG mutations can impair adipogenesis, reduce insulin sensitivity, and promote severe metabolic disease. Early functional studies identified mutations in both the DNA-binding domain (DBD) and LBD of PPARγ that cause lipodystrophic insulin resistance. Agostini et al. described human PPARγ mutants, including V290M, R425C, and P467L, that lacked normal transcriptional activity and exerted dominant-negative effects on the wild-type receptor [58]. Monajemi et al. later reported the R194W mutation in the DBD of PPARγ2, showing that this variant disrupts DNA binding and causes familial partial lipodystrophy through loss of receptor function [59]. These studies were not MD papers, but they defined the biological context for later structural and computational work on PPARγ mutations.

One of the clearest structure-based analyses of a disease-associated PPARγ mutation is the study of the F360L mutant, corresponding to residue F388 in PPARγ2, which is associated with familial partial lipodystrophy. Lori et al. combined crystallography, spectroscopy, calorimetry, transactivation assays, and MD to explain the transactivation deficiency of this mutant [60]. The F360 residue is in the LBD and contributes to the structural integrity of the receptor. Substitution by leucine altered the receptor’s response to agonists and reduced transcriptional activation. The integration of structural and dynamic analyses showed that disease-associated substitutions can affect receptor function not only by directly disrupting ligand contacts but also by modifying the stability and communication properties of the LBD.

A broader view of PPARγ disease mutations was provided by Petrosino et al., who examined several nonsynonymous single-nucleotide polymorphisms of the PPARγ LBD, including variants associated with cancer, lipodystrophy, and insulin resistance [61]. The authors investigated V290M, R357A, F360L, P467L, Q286P, R288H, E324K, E460K, and related variants using a combination of biochemical, spectroscopic, and MD approaches. Their results indicated that different mutations can perturb receptor stability, ligand responsiveness, and local structural flexibility in distinct ways. This work shows why sequence position alone is not enough to predict the pathological effect of a PPARγ variant. Instead, disease-associated mutations must be interpreted in the context of the three-dimensional fold, the ligand-binding pocket, AF-2 dynamics, and long-range communication pathways.

Computational screening approaches have also been used to prioritize potentially deleterious PPARγ variants. Stalin et al. performed an in silico analysis of single-nucleotide polymorphisms in PPARγ associated with obesity, diabetes, and cancer [62]. After identifying potentially damaging variants, they introduced mutations such as C162S, R166W, Q286P, Q314P, and P467L into structural models and used MD simulations to evaluate their dynamic stability and flexibility. Trajectory analyses, including RMSD, RMSF, and radius of gyration, suggested that these variants altered the structural behavior of PPARγ compared with the wild-type receptor. Although such studies are less experimentally integrated than crystallography- or NMR-supported analyses, they are useful for prioritizing variants and generating hypotheses about how disease-associated mutations may perturb receptor conformational stability.

The P12A polymorphism in PPARγ2 represents a different type of disease-associated variant. Unlike rare FPLD3 mutations, P12A is a common polymorphism that has been studied extensively in relation to type 2 diabetes, cardiovascular disease, obesity, and nonalcoholic fatty liver disease. Taghvaei and Saremi used molecular modeling, stability-prediction tools, MD simulations, and essential dynamics to examine the structural consequences of the P12A substitution in PPARγ2 [63]. Their simulations suggested that the mutation affects the stability and flexibility of the modeled PPARγ2 structure, with changes in RMSD, RMSF, radius of gyration, solvent-accessible surface area, hydrogen bonding, secondary structure, and essential motions. This study is relevant because it extends MD analysis beyond the LBD and highlights the potential contribution of N-terminal variants to disease-associated receptor behavior. However, interpretation of P12A simulations requires caution because the N-terminal A/B region of PPARγ2 is intrinsically flexible and less structurally constrained than the LBD.

Recent work has also shown that disease-associated PPARγ mutations can disrupt protein-protein and protein-DNA interfaces rather than only ligand binding. Madsen et al. investigated two lipodystrophy-associated PPARγ mutations, R212Q and E379K, located in the hinge region and LBD, respectively [64]. These variants were predicted to interfere with distinct interfaces in the PPARγ-RXR-DNA complex. In detail, R212Q affects interaction with the DNA minor groove near the 5′ extension of the PPRE, whereas E379K disrupts an interface between the PPARγ LBD and the RXRα DBD. Using biochemical, genome-wide, structural, and modeling approaches, the authors showed that both mutations impair activation of a subset of PPARγ target enhancers and reduce adipogenic capacity. For an MD-oriented review, the main value of this study is that it shifts the mutation problem from isolated LBD stability to the stability of multidomain PPARγ-RXR-DNA assemblies.

Compared with PPARγ, disease-associated PPARα variants have received less atomistic MD attention. PPARα polymorphisms, including L162V and V227A, have been associated with lipid metabolism, dyslipidemia, atherosclerosis risk, and metabolic phenotypes [65]. However, most work on these variants remains genetic, epidemiological, or functional, rather than structural-dynamic. This represents an important gap because PPARα regulates fatty acid oxidation and lipid catabolism, and disease-associated variants could plausibly alter ligand sensitivity, AF-2 dynamics, RXR communication, or transcriptional responses. Similar limitations apply to PPARβ/δ, for which disease associations have been proposed in metabolic disease, inflammation, cancer, and cardiovascular biology, but detailed MD studies of pathogenic or clinically relevant variants remain scarce.

**Figure 3:**
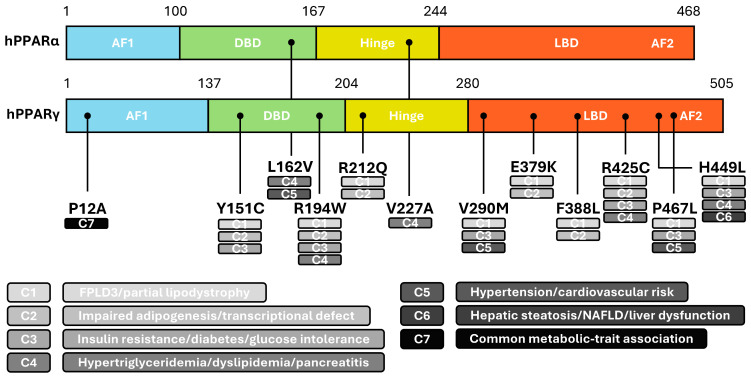
Schematic representation of the disease-associated mutations with clinical evidence, mapped to their approximate positions along the receptor sequence. Each mutation is annotated with one or more disease/phenotype categories: **C1**, familial partial lipodystrophy type 3 (FPLD3)/partial lipodystrophy; **C2**, impaired adipogenesis or transcriptional defect; **C3**, insulin resistance, diabetes, or glucose intolerance; **C4**, hypertriglyceridemia, dyslipidemia, or pancreatitis; **C5**, hypertension or cardiovascular risk; **C6**, hepatic steatosis, NAFLD, or liver dysfunction; and **C7**, common metabolic-trait association.

The current literature suggests several ways in which PPAR mutations can impair receptor function. Some variants disrupt DNA binding or PPRE recognition, while others alter ligand-binding pocket geometry, LBD stability, H12/AF-2 behavior, co-regulator recruitment, RXR interfaces, or phosphorylation-linked regulatory regions. A schematic representation of the disease-associated mutations is shown in Figure 3. MD simulations are particularly useful for identifying dynamic consequences that are not evident from static structures, including changes in flexibility, correlated motions, stability, solvent exposure, and long-range communication. A summary of studies on PPAR disease-associated mutations and structural/dynamic mechanisms is shown in Table S3—SM.

## 4. PPAR–Ligand Interactions: Molecular Dynamics Perspectives

Ligand binding is the central event linking PPAR structure to biological function. The PPAR LBD contains a large, branched, Y-shaped cavity that can accommodate many chemically different ligands, including endogenous fatty acids, fibrates, thiazolidinediones, partial agonists, antagonists, inverse agonists, phytochemicals, cannabinoids, and dual or pan-PPAR modulators. Because ligand binding can affect H12, AF-2, the Ω-loop, co-regulator recruitment, and distal allosteric pathways, MD has become an essential tool for understanding PPAR pharmacology beyond static docking or crystallographic poses. A summary of MD-focused studies on PPAR–ligand interactions is shown in Table S4—SM.

### 4.1. Ligand Entry, Residence, and Escape Pathways

One of the earliest MD questions in the PPAR field concerned how ligands enter and exit the deeply buried LBD cavity. Genest et al. used MD simulations to investigate ligand-escape pathways from the PPARγ LBD, showing that ligand dissociation can occur through multiple routes rather than through a single rigid opening [66]. The main message from this early work was that the PPARγ pocket behaves as a fluctuating cavity, not as a rigid binding site.

Aci-Sèche et al. later used targeted MD to investigate ligand entry pathways for the flexible ionic partial agonist GW0072 in PPARγ [67]. Starting from the ligand outside the receptor, the simulations produced docked conformations close to the holo complex and suggested that entry is guided largely by hydrophobic interactions. Importantly, the entry routes resembled previously described exit routes, supporting the idea that ligand association and dissociation are governed by reversible dynamic pathways. These studies anticipated later work showing that PPARγ ligands may bind through transient encounter complexes before reaching the orthosteric pocket [37]. Therefore, ligand efficacy cannot be understood only from the final bound pose. Association pathways, intermediate states, and pocket breathing may also influence residence time, selectivity, and functional output.

### 4.2. Endogenous Fatty Acids and Natural Lipid Ligands

Endogenous lipids are physiologically relevant PPAR ligands and provide an important reference for interpreting synthetic modulators. Medium-chain fatty acids were shown to act as selective PPARγ activators and pan-PPAR partial agonists [68]. Structural analysis, B-factor interpretation, and MD simulations suggested that these fatty acids stabilize H12 more weakly than classical thiazolidinedione full agonists and that their effects depend strongly on chain length [68]. This finding fits well with the broader model of partial agonism, in which ligands can activate PPARs without fully locking H12 in the canonical active conformation.

**Figure 4:**
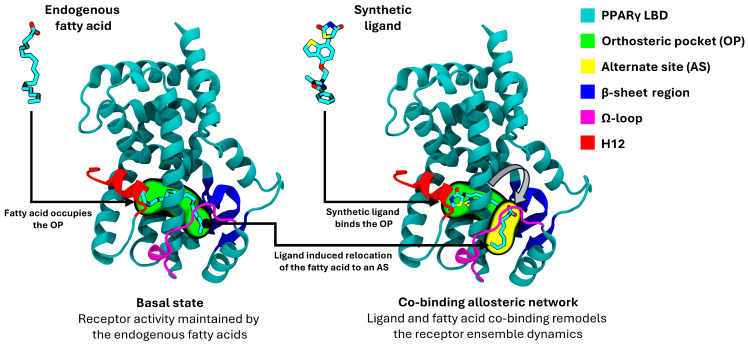
Schematic representation of cooperative co-binding between an endogenous fatty acid and a synthetic ligand in the PPARγ ligand-binding domain. PPARγ is shown as a cyan cartoon; the orthosteric pocket (OP) and alternate site (AS) are highlighted in green and yellow, respectively, while the β-sheet region, Ω-loop, and helix 12 (H12) are shown in blue, magenta, and red. An endogenous fatty acid occupies the OP and contributes to maintenance of the receptor conformational ensemble (**left figure**). Subsequent binding of a synthetic ligand to the OP does not necessarily eject the fatty acid from the receptor. Instead, the synthetic ligand occupies the OP and promotes relocation of the fatty acid toward the adjacent AS at the β-sheet/Ω-loop interface, enabling simultaneous ligand and fatty acid occupancy (**right figure**). The arrows indicate ligand entry and the proposed direction of fatty-acid relocation. Cooperative occupancy of the OP and AS can remodel coupling among the β-sheet region, H3, the Ω-loop, and H12/AF-2, thereby redistributing the PPARγ conformational ensemble and potentially altering co-regulator recruitment and transcriptional output [35].

The ability of PPARγ to accommodate multiple lipidic ligands was further demonstrated by the discovery of an alternate ligand-binding site [69]. Hughes et al. identified a secondary site near the Ω-loop that can bind ligands outside the canonical orthosteric pocket. This was later extended by cooperative co-binding studies showing that synthetic ligands can displace endogenous fatty acids from the orthosteric cavity into an adjacent site near the Ω-loop [35]. As illustrated in Figure 4, synthetic-ligand binding can occupy the orthosteric pocket while relocating a pre-bound fatty acid to the adjacent alternate site at the β-sheet/Ω-loop interface, thereby permitting simultaneous occupancy of both sites. This co-binding arrangement may remodel coupling among the β-sheet region, H3, the Ω-loop, and H12/AF-2, with potential consequences for co-regulator recruitment and transcriptional output [35]. PPARγ ligand pharmacology may therefore involve multi-ligand occupancy, intra-pocket relocation, and allosteric communication between the orthosteric cavity and the β-sheet/Ω-loop region.

For PPARα, Kamata et al. solved a large series of human PPARα LBD structures bound to endogenous fatty acids and fibrates and complemented the structural analysis with MD, mutagenesis, and functional assays [70]. Their work identified stearic acid and palmitic acid as plausible physiological PPARα ligands and showed that ligand coordination in specific pocket regions is important for potency and selectivity. These results give an isoform-specific structural and dynamic basis for understanding why PPARα responds strongly to fatty-acid-like ligands and fibrate drugs.

### 4.3. Full Agonists, Partial Agonists, and Graded Activation

The distinction between full and partial agonism is one of the central themes in PPAR ligand biology. Classical PPARγ full agonists such as rosiglitazone and pioglitazone stabilize H12 through interactions with the polar arm of the binding pocket, thereby favoring formation of the AF-2 coactivator surface [14,15,16]. In contrast, partial agonists frequently stabilize H3, the β-sheet region, or alternative pocket regions without forming the canonical H12 stabilizing interaction [16,34]. MD studies are particularly valuable for this problem because structures of full and partial agonist complexes can appear similar, even when their effects on conformational ensembles and transcriptional activity are different. In this context, rationally designed glitazone derivatives have been evaluated through integrated in silico workflows combining docking and MD simulation, demonstrating that the latter can support the optimization of synthetic PPARγ agonists before biological validation [71,72]. More recently, Haider et al. integrated ML with in silico drug design to uncover new PPARγ agonists, using computational filtering, docking, MD simulation, and binding-energy analysis to prioritize candidate compounds [73].

Capelli et al. identified a novel PPAR pan-agonist and showed that alternative ligand-binding modes can produce partial or full activation depending on the receptor context [34]. In PPARα, the ligand occupied a new pocket whose formation involved ligand-induced rearrangement of a phenylalanine side chain, whereas in PPARγ different binding features contributed to activation. In this connection, PPAR ligand efficacy depends not only on whether the ligand binds but also on which sub-pocket it occupies, which receptor elements it stabilizes, and how it reshapes the AF-2 ensemble. Indeed, a recent microsecond-scale MD study of pemetrexed, a first line non-small-cell lung cancer drug, provided an atomistic basis for its recognition as a PPARγ partial agonist, showing that pemetrexed adopts a binding mode that avoids direct AF-2 engagement while inducing PCA, free-energy landscape, and correlated-motion patterns consistent with partial agonism [74].

Long-timescale MD comparisons of ligands across PPAR isoforms have provided additional insight into graded activation. Sullivan et al. simulated the pan-agonist chiglitazar bound to PPARα, PPARβ/δ, and PPARγ for 3 μs per complex [39]. MM-GBSA calculations predicted the strongest binding to PPARγ, followed by PPARα and PPARβ/δ, consistent with experimental trends. Residue-level energy decomposition and interaction analysis showed that pan-agonism reflects a balance between conserved AF-2 stabilization and isoform-specific ligand accommodation. The study also highlighted that H12 conformation and flexibility are key determinants distinguishing full and partial activation. In contrast to the microsecond-scale and ensemble-oriented studies discussed above, the MD and MM-GBSA analysis of kaempferol, quercetin, and resveratrol derivatives mainly provides screening-level evidence that selected polyphenol scaffolds can form stable PPARγ complexes and engage the LBD through nonclassical interaction networks [75].

Recent ensemble-based work further reinforces this interpretation. Studies of PPARγ conformational ensembles show that ligand efficacy correlates with redistribution of receptor populations rather than with stabilization of one static structure [21,23]. A related strategy was later applied to PPARγ by Prabitha et al., who generated multi-conformational pharmacophore models from MD trajectories of a rosiglitazone-bound PPARγ complex [76]. One of the most methodologically relevant examples is the MD-shared pharmacophore strategy developed for PPARα by Perricone et al. [77]. By extracting representative frames from the simulation, the authors converted a single crystallographic complex into an ensemble of receptor conformations suitable for mapping and screening potential ligands [77].

Virtual screening workflows have also been applied to identify PPARγ agonists and antidiabetic candidates from larger chemical libraries. However, these studies should be interpreted primarily as hit-prioritization pipelines unless they include functional validation or analyses that connect ligand binding to receptor conformational redistribution. Ahmed et al. combined virtual screening, MD, density functional theory, and QSAR modeling to design PPARγ agonists as potential antidiabetic agents [78]. In a similar way, Mandal et al. have applied a virtual screening approach combined with MD and in vitro validation to find agonist compounds for PPARβ/δ [79]. These examples illustrate a useful stratification of the MD framework. High-throughput docking and short MD can narrow large chemical libraries, whereas mechanistic conclusions require deeper simulations on prioritized hits, ideally including longer trajectories, enhanced sampling, ensemble analysis, and experimental validation.

### 4.4. Dual and Pan-PPAR Agonists

Dual and pan-PPAR agonists are pharmacologically attractive because they can combine metabolic effects mediated by different isoforms. However, they also pose a structural challenge, since the ligand must interact productively with related but non-identical LBD pockets. In this area, MD has been used in two different ways: as a post-docking stability filter for newly proposed dual/pan candidates and as a mechanistic tool to compare how established ligands engage multiple PPAR isoforms.

Early structure-based pharmacophore and MD work on traditional Chinese medicine compounds proposed berberrubine and (S)-tryptophan-betaxanthin as potential “three-in-one” PPARα/γ/δ agonists, illustrating how MD was already being used to refine docking-derived pan-PPAR binding hypotheses [80]. High-throughput virtual screening combined with docking and MD simulations has also been applied to pharmacological ligand libraries to identify potential pan-PPAR agonists, reinforcing the value of MD-based stability assessment for ligands intended to modulate PPARα, PPARβ/δ, and PPARγ simultaneously [81]. Recently, Mandal et al. proposed polydatin as a pan-PPAR agonist for NAFLD through a workflow combining high-throughput virtual screening, docking/MD analysis, and in vitro validation in a HepG2 steatosis model, illustrating the translational potential of MD-supported pan-PPAR ligand discovery [82]. The translational relevance of pan-PPAR modulation is illustrated by a recent bezafibrate study in experimental rheumatoid arthritis, where computational prioritization was followed by in vivo anti-inflammatory validation [83].

Liu et al. combined virtual screening, ADMET prediction, and MD simulations to identify ASN15761007 as a potential PPARα/γ dual agonist [84]. Their MD simulations suggested that ASN15761007 adopted favorable binding conformations in both receptors, resembling the activation behavior of known PPARα and PPARγ agonists. Similarly, various computational methods integrated with MD were also used to identify CHEMBL230490 as a potential PPARα/γ dual agonist [85]. Structure-based docking and MD simulations of PPARα/γ dual agonist candidates further showed that dual activity depends on maintaining stable interaction patterns across both receptor pockets [86]. These studies are representative of a large class of ligand-discovery papers in which MD is used after docking to test complex stability. Their mechanistic value depends on whether the simulations go beyond pose persistence to compare isoforms, quantify residue-level energetic differences, or connect binding to AF-2/H12 behavior.

Chiglitazar represents a more mechanistically characterized pan-PPAR example [39]. As discussed above, the comparative 3 μs simulations of chiglitazar with all three PPAR isoforms provided a direct MD-based framework for understanding pan-agonism. Importantly, such isoform-matched simulations are more informative than isolated single-receptor studies because they reveal which interactions are conserved and which are isoform-specific. This type of comparative MD design should become standard for evaluating dual and pan-PPAR ligands.

### 4.5. Natural Products and Phytochemical PPAR Modulators

Natural products are widely investigated as PPAR modulators because they offer chemically diverse scaffolds and may produce partial agonism or selective modulation with fewer adverse effects than classical full agonists. Many such studies use docking followed by relatively short MD simulations to test whether predicted ligand poses remain stable inside the PPAR pocket [87]. This strategy is valuable for excluding unstable poses and prioritizing compounds, but it should not be equated with computational proof of agonism, partial agonism, or selective modulation.

Phytocannabinoids provide a useful example of how confidence increases when docking/MD predictions are combined with biological validation. D’Aniello et al. identified cannabigerolic acid, cannabidiolic acid, and cannabigerol as dual PPARα/γ agonists using a combined computational and experimental approach [88]. Similarly, Iannotti et al. identified cannabimovone as a cannabis-derived PPARγ agonist through docking, 50 ns MD simulations, and functional experiments [89]. In both cases, the functional assays are essential, because short MD mainly supports binding-pose stability rather than proving transcriptional activity. Moreover, Citrus-derived eriocitrin and its metabolites were also proposed as PPARγ partial agonists using docking and MD simulations, adding nutraceutical flavonoid metabolites to the expanding class of natural compounds predicted to stabilize PPARγ without classical full-agonist behavior [90]. In screening Nelumbinis folium compounds against PPARγ, MD/essential-dynamics analysis pointed out narcissin as a promising weight-management candidate, based on its stable pocket interactions and favorable binding energetics [91]. For natural ligands that differ chemically from classical synthetic agonists, MD can help identify which pocket contacts and dynamic responses make the predicted activity plausible [92].

Natural compounds have also been explored as PPARγ modulators in inflammatory bowel disease, with docking and MD simulations used mainly to prioritize ligands capable of maintaining stable interactions within the PPARγ ligand-binding pocket [93]. In disease-oriented studies, neferine provides a stronger example because docking/MD evidence for stable PPARγ binding was complemented by animal experiments supporting a PPARγ-dependent anti-inflammatory mechanism in a depression model [94]. By contrast, computational screening of polyphenolic scaffolds against PPARα, using docking, MD, and MM/GBSA, mainly identifies kaempferol, resveratrol, and quercetin derivatives as stable LBD binders and candidate natural product-inspired scaffolds [95]. Similarly, PPARα-focused screening of Solanum erianthum phytoconstituents suggests that apigenin can form a comparatively stable PPARα complex, but this remains a ligand-prioritization result unless linked to receptor activation, isoform selectivity, or experimental validation [96].

Other phytochemical-oriented studies have used MD to compare candidate natural products or derivatives in the PPARγ pocket. Muralikumar et al. performed docking, binding free-energy calculations, and MD simulations to rank bioactive lipids interacting with PPARγ [97]. More recently, Lian et al. used virtual screening of a natural-product library, MD simulation, and MM-PBSA analysis to identify potential PPARγ partial agonists that do not directly interact with the AF-2 helix [98]. Such studies are useful because they search for compounds capable of stabilizing non-canonical LBD regions, but their strongest role is the generation of promising candidates. Confirmation of partial agonism still requires evidence that these ligands redistribute H12/AF-2 or β-sheet dynamics in a way consistent with structural, HDX-MS, or functional data [16,34].

### 4.6. Antagonists, Inverse Agonists, and Repressive Ligand Mechanisms

Compared with agonists and partial agonists, PPAR antagonists and inverse agonists are less commonly studied by MD, but they are crucial for understanding repression and selective modulation [99]. Earlier enhanced-sampling studies showed that apo PPARγ can access antagonist-like H12 states [19], while later structural and biophysical studies captured repressive ligand-bound conformations [33]. The outcome of these studies indicates that antagonism and inverse agonism actively remodel H12 and corepressor-compatible ensembles, rather than merely lacking agonist stabilization.

Recent work has identified structural determinants of non-covalent PPARγ inverse agonism [100]. Kuo et al. combined structural analysis, MD simulations, and functional evaluation to examine how non-covalent ligands can drive repressive PPARγ states [100]. The repression of PPARγ can also open new roadmaps for developing novel anti-obesity pharmacotherapies. Indeed, topiramate-phenolic acid conjugates were designed as PPARγ inhibitors using QSAR, docking, and MD simulation followed by biological evaluation, supporting the emerging interest in PPARγ inhibition as an anti-obesity strategy [101]. These studies show that PPARγ repression can be actively ligand-stabilized rather than interpreted as a simple absence of agonism. NMR-guided ligand-series work by MacTavish et al. further strengthens this view by showing that small chemical changes can shift PPARγ behavior across an inverse agonist to agonist spectrum, with ligand function tracking redistribution between active-like and repressive-like substates [102]. This work provides a direct experimental framework for interpreting MD simulations as attempts to quantify ligand-dependent population shifts across pre-existing receptor states.

Newer partial-agonist discovery studies also point toward noncanonical pockets and alternative mechanisms. Wang et al. reported a novel binding pocket in PPARγ for partial agonists and used MD simulations to evaluate the stability of the PPARγ-TWSZ-5 complex [103]. Similarly, Liu et al. used an integrated workflow combining fragment molecular orbital calculations, ML, docking, interaction-fingerprint filtering, and MD simulations to identify PPARγ partial agonist candidates for type 2 diabetes [104]. These studies support the possibility that selective modulation can arise from nonclassical pockets or conformational states outside the canonical full-agonist mechanism, but the strength of this conclusion depends on whether the MD analysis captures ensemble redistribution rather than only ligand-pose persistence.

### 4.7. General Lessons from MD Studies of PPAR–Ligand Interactions

**Figure 5:**
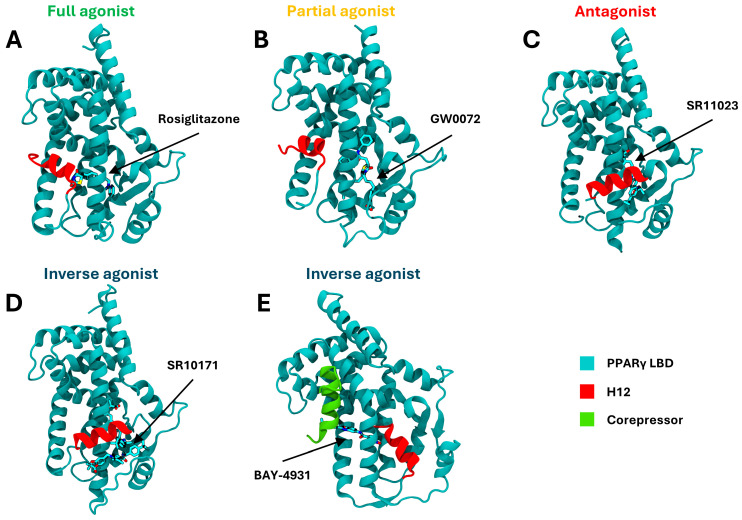
Representative structural states of the PPARγ ligand-binding domain (LBD) associated with full agonist, partial agonist, antagonist, and inverse agonist. The PPARγ LBD is shown as a cyan cartoon, bound ligands are shown as sticks, and helix 12 (H12) is highlighted in red. (**A**) Full agonist bound PPARγ with rosiglitazone (PDB ID: 7AWC [108]). Rosiglitazone stabilizes H12 in the canonical activation function-2 (AF-2)-competent position, promoting formation of the coactivator-binding surface, robust coactivator recruitment, and strong transcriptional activation. (**B**) Partial agonist bound PPARγ with GW0072 (PDB ID: 4PRG [109]). GW0072 binds in an alternative region of the ligand-binding pocket, and does not efficiently stabilize H12, which retains a more apo-like or conformationally heterogeneous arrangement. This state is associated with weak or incomplete AF-2 stabilization, reduced or selective coactivator recruitment, and graded transcriptional activation. (**C**) Antagonist bound PPARγ with SR11023 (PDB ID: 6C5T [110]). SR11023 stabilizes a non-active H12 orientation displaced from the canonical AF-2 position, thereby blocking agonist-dependent coactivator recruitment while permitting corepressor-compatible conformations without necessarily reducing basal receptor activity. (**D**) Non-covalent inverse agonist bound PPARγ with SR10171 (PDB ID: 6C5Q [110]). SR10171 repositions H12 toward the ligand-binding pocket and shifts the receptor toward repressive conformational states, favoring corepressor compatibility and decreasing basal transcriptional activity. (**E**) Covalent inverse agonist bound PPARγ with BAY-4931 and an NCOR2 corepressor peptide (PDB ID: 8AQN [111]). BAY-4931 is covalently attached to Cys285, while H12 is packed into the ligand-binding pocket and the corepressor peptide, shown in green, occupies the repressive co-regulator-binding surface. This arrangement strongly stabilizes the repressive ensemble and suppresses basal transcriptional activity. Together, the structures illustrate a progression from robust AF-2 stabilization and activation to graded activation, antagonism, and increasingly stabilized repressive states.

Within the extensive literature on PPAR–ligand interactions, several key themes consistently emerge. First, ligand binding to PPARs is dynamic and may involve entry pathways, escape routes, encounter complexes, and internal migration between sub-pockets [66,67,68,69]. Second, ligand efficacy is not determined only by binding affinity. Full agonists, partial agonists, inverse agonists, and antagonists reshape the conformational ensemble differently, particularly around H12, AF-2, H3, the β-sheet, and the Ω-loop [16,19,21,22,23,33,34]. A qualitative representation of PPARγ associated with full agonist, partial agonist, antagonist, and inverse agonist is shown in Figure 5. Third, isoform selectivity depends on both conserved activation motifs and subtle differences in pocket geometry, polarity, and flexibility [29,30,31,32,39,70]. Fourth, docking alone is insufficient for mechanistic interpretation. Short MD can improve docking by testing pose persistence, interaction stability, and overall pocket compatibility, but mechanistic interpretation requires additional evidence, such as free-energy analysis, residue decomposition, ensemble redistribution, dynamic-network analysis, longer or enhanced-sampling simulations, and ideally experimental validation.

Multitarget ligand-discovery workflows are another emerging direction. Kaya et al. screened compounds against both PPARγ and α-glucosidase using molecular docking, MD simulations, and MM/PBSA analysis to identify potential dual-target antidiabetic agents [105]. A similar multitarget antidiabetic workflow was recently applied to plant-derived compounds against PPARγ, 11β-HS1, DPP4, and SGLT2, confirming how MD is increasingly used to filter docking-derived multitarget hypotheses [106]. This approach is conceptually distinct from classical single-target PPAR modulation because the desired compound must satisfy interaction requirements in two unrelated binding pockets. A major limitation of the field is that many PPAR ligand-discovery studies use short MD simulations primarily as post-docking validation. Such simulations are useful for screening because they can discard unstable complexes and identify persistent interactions, but they rarely capture slow H12 rearrangements, ligand migration, AF-2 ensemble redistribution, ligand residence-time effects, or co-regulator-dependent conformational changes [107]. Future work should therefore prioritize longer simulations, enhanced sampling, ligand residence-time estimation, matched isoform comparisons, and simulations including RXR, DNA, and co-regulator peptides.

## 5. Discussion and Future Perspectives

The MD literature on PPARs has progressively moved from static interpretations of ligand binding toward a more integrated view in which receptor function emerges from conformational ensembles, ligand chemistry, dimerization state, co-regulator recruitment, disease-associated mutations, and post-translational modifications. The central conclusion of this review is that PPAR activity should not be interpreted only as the consequence of ligand binding to an isolated ligand-binding domain. Instead, PPARs should be understood as dynamic transcriptional platforms whose biological output depends on the coupling between chemical signals, protein–protein interactions, DNA response elements, and cell-specific regulatory networks.

### 5.1. From Ligand Binding to Context-Dependent Transcriptional Regulation

Most MD studies on PPARs still focus on isolated ligand-binding domains, especially PPARγ, and often use docking-derived complexes as the starting point for simulations. These studies are informative for ligand pose stability, hydrogen-bond persistence, H12 dynamics, AF-2 stabilization, and subtype-selective pocket interactions, but they are not necessarily mechanistically explanatory in the transcriptional sense. This level of analysis captures only part of PPAR biology because it usually omits RXR, DNA, co-regulators, PTMs, and the multidomain communication pathways that determine receptor output. In the cellular context, PPARs operate as multidomain transcription factors that interact with RXR, DNA, coactivators, corepressors, kinases, and chromatin-associated proteins [112]. Therefore, the biological meaning of ligand binding depends on how local ligand-induced perturbations propagate across larger transcriptional assemblies.

This point is particularly evident from studies of PPAR-RXR complexes. Structural and computational analyses of PPARγ-RXRα and PPARα-RXRα demonstrated that ligand binding, heterodimerization, DNA recognition, and co-regulator recruitment are coupled through allosteric communication pathways [26,28,43,47]. These studies indicate that the receptor complex behaves as an integrated dynamic unit rather than as two independent ligand-binding proteins. Consequently, future MD studies should increasingly move beyond isolated LBD simulations and include RXR, DNA response elements, coactivator or corepressor peptides, and relevant post-translational states.

This shift is important not only for structural accuracy but also for pharmacological interpretation. A ligand that appears stable in an isolated LBD may have different behavior in a full PPAR-RXR-DNA structure. Within this complex, the same binding event can affect dimerization geometry, DNA-binding domain communication, co-regulator affinity, and transcriptional polarity. Therefore, ligand efficacy should be evaluated not only through binding energy or ligand RMSD but also through its effect on receptor-wide communication networks.

### 5.2. Ligand Chemistry as Conformational State Selection

The chemical diversity of PPAR ligands is one of the most distinctive features of this receptor family. Endogenous fatty acids, fibrates, thiazolidinediones, partial agonists, inverse agonists, natural products, cannabinoids, and dual or pan-PPAR agonists all engage the same general LBD architecture, but they do not necessarily stabilize the same functional state. The emerging picture from structural biology, MD simulations, HDX-MS, NMR, and co-regulator assays is that ligand efficacy is better described as conformational-state selection than as a simple active/inactive switch.

In this framework, full agonists preferentially stabilize H12 and the AF-2 coactivator surface, whereas many partial agonists stabilize the β-sheet region, H3, or alternative pocket regions without completely locking H12 in the canonical active position [16,21]. Inverse agonists and antagonists, in contrast, may promote repressive H12 conformations or corepressor-compatible states [22,33]. Thus, two ligands with comparable docking scores, or even comparable short-MD stability, may have very different biological effects if they redistribute the receptor ensemble differently. Computational studies should therefore not focus only on whether a compound binds, but on whether it stabilizes an agonist-like, partial-agonist-like, antagonist-like, or corepressor-compatible substate. For each new ligand, simulations should evaluate H12 mobility, AF-2 geometry, β-sheet and H3 stabilization, Ω-loop dynamics, ligand migration between subpockets, and long-range communication toward RXR and DNA-binding interfaces. For dual and pan-PPAR ligands, matched simulations across PPARα, PPARβ/δ, and PPARγ should become standard, because isoform selectivity depends on slight differences in pocket geometry, residue-level contacts, and dynamic flexibility.

### 5.3. Disease-Associated Mutations as Natural Perturbations of PPAR Allostery

MD simulations offer a powerful approach for investigating disease-associated PPAR mutations at a mechanistic level. In particular, pathogenic PPARG variants linked to familial partial lipodystrophy, insulin resistance, diabetes, dyslipidemia, and impaired adipogenesis demonstrate that relatively local sequence changes can disrupt global receptor function [53,54,55,56,57,58,60,64]. Some mutations directly affect DNA binding or PPRE recognition, whereas others perturb ligand-binding pocket stability, AF-2 dynamics, RXR interfaces, or multidomain transcriptional assemblies.

The current literature shows that mutation-focused MD is much less developed than ligand-focused MD. The few available MD studies already indicate that disease variants can act through distinct mechanisms. The F360L mutation (F388L in PPARγ2) illustrates how a lipodystrophy-associated LBD mutation can reduce transactivation by altering receptor stability and ligand responsiveness, rather than by simply disrupting one local contact [60]. Broader MD-supported analyses of PPARγ variants, including V290M, R357A, F360L, P467L, Q286P, R288H, E324K, and E460K, further suggest that different substitutions can perturb LBD stability, flexibility, and ligand-dependent activation through variant-specific mechanisms [61]. Similarly, computational prioritization studies of variants such as C162S, R166W, Q286P/Q314P, and P467L show that MD can help identify changes in global stability and local flexibility, although such predictions require experimental validation [62]. The P12A polymorphism extends this perspective beyond the LBD, suggesting that common N-terminal variants may also alter receptor flexibility, even though simulations of this intrinsically flexible region should be interpreted cautiously [63].

Several clinically important PPARG mutations should now become priorities for more mechanistic MD. The dominant-negative P467L and V290M mutations were among the first variants linked to severe insulin resistance, diabetes mellitus, and hypertension, and structural interpretation suggests that they impair transactivation by destabilizing the activation surface, particularly around H12 [57,113]. R425C is another strong candidate because MD predicted disruption of a conserved salt-bridge network and formation of an alternative salt bridge, linking electrostatic rearrangement to impaired receptor function [114]. These LBD mutations should be studied with enhanced sampling in apo, agonist-bound, partial-agonist-bound, and co-regulator-bound states to determine whether they shift the free-energy balance between active, partial, and repressive ensembles.

Moreover, R194W in the PPARγ2 DNA-binding domain disrupts DNA binding and causes familial partial lipodystrophy, making it a logical target for simulations of PPARγ-RXRα-PPRE complexes [59]. Similarly, R212Q and E379K impair enhancer activation by perturbing PPARγ-DNA and PPARγ-RXR-related interfaces, respectively, and therefore cannot be fully understood using isolated LBD simulations [64]. Recent clinical reviews of FPLD3 report substantial genetic and phenotypic heterogeneity, including many pathogenic or likely pathogenic PPARG variants, reinforcing the need for systematic structural prioritization of mutations for MD studies [53,54]. Newly described DBD variants, such as Y151C, further support the importance of modeling DNA-bound receptor complexes rather than focusing only on ligand-pocket mutations [115].

Expanding beyond PPARγ, PPARα variants such as L162V and V227A have been associated with lipid metabolism, dyslipidemia, atherosclerosis risk, and metabolic phenotypes. However, their effects on ligand recognition, H12 stabilization, fibrate response, and PPARα-RXRα communication remain poorly characterized [65]. Comparable atomistic studies are even scarcer for PPARβ/δ, despite its roles in lipid metabolism, inflammation, skeletal muscle function, wound healing, and cancer biology. Future work should therefore treat disease mutations not only as clinical annotations but also as mechanistic probes. In detail, LBD mutations should be tested for effects on ligand-dependent ensemble redistribution, DBD and hinge mutations in DNA-bound PPAR-RXR complexes, interface mutations in multidomain assemblies, and common polymorphisms in models that account for flexible domains and co-regulator recruitment.

### 5.4. Post-Translational Modifications as Dynamic Switches

Post-translational modifications (PTMs) represent another major underexplored frontier for PPAR MD. PPAR functions are regulated not only by ligand binding but also by phosphorylation, SUMOylation, ubiquitination, acetylation, O-GlcNAcylation, and other modifications [116,117]. These modifications can alter receptor stability, subcellular localization, co-regulator recruitment, transcriptional activity, degradation, and disease-associated signaling. From a structural-dynamic perspective, PTMs should be viewed as allosteric perturbations capable of rewiring receptor communication networks.

The best-established example is Cdk5-mediated phosphorylation of PPARγ at S273 in PPARγ2, corresponding to S245 in PPARγ1. This modification is associated with obesity-linked insulin resistance and can be modulated by antidiabetic ligands that do not necessarily act as classical full agonists [50,118]. MD and integrative studies have shown that phosphorylation can alter collective motions in the PPARγ-RXRα-DNA complex and that ligand binding can inhibit phosphorylation by stabilizing regions near the β-sheet and Ω-loop [47,51,52]. Together, these data support the broader concept that therapeutic ligands may act not only by activating transcription but also by modulating the dynamic accessibility of PTM-regulated surfaces.

Moving forward, other PTMs deserve similar attention for their biological impact. Acetylation and deacetylation of PPARγ, including SIRT1-dependent regulation, have been linked to adipocyte browning and metabolic remodeling [119]. Recent modeling and integrative studies have begun to extend MD-based PTM analysis beyond phosphorylation alone. Malospirito et al. modeled SIRT1 recognition of PPARγ acetylated at K268 and K293 and proposed that the SIRT1 N-terminal domain provides a common anchoring interface for acetylated PPARγ states [120]. A subsequent study further showed that K268/K293 acetylation and S273 phosphorylation are functionally interconnected: acetyl-mimetic K268/K293 substitutions reduced CDK5 binding and phosphorylation while enhancing transcriptional activity, while S273 phosphorylation weakened SIRT1 binding [121]. These findings suggest that PPARγ PTMs should be considered as combinatorial allosteric regulators that reshape kinase, deacetylase, and co-regulator accessibility, rather than as isolated local chemical modifications.

SUMOylation and ubiquitination also contribute to transcriptional repression, receptor turnover, inflammatory signaling, and metabolic control [116,117]. However, few MD studies explicitly model these modified states [122]. Future work would benefit from more MD simulations focused specifically on SUMOylated and ubiquitinated PPARs, including comparisons with unmodified receptors and analyses of how these PTMs interact with ligand class.

### 5.5. Dimerization Beyond the Canonical PPAR-RXR Model

PPARs are classically described as RXR heterodimeric transcription factors, and the PPAR-RXR complex remains the physiologically dominant model for PPAR-dependent transcription [26,123,124]. Nevertheless, the broader nuclear receptor field shows that dimerization is more diverse than a single canonical partnership [2]. Nuclear receptors can form homodimers or heterodimers, and RXR itself is a central hub that forms multiple permissive and non-permissive heterodimers with nuclear receptor partners [123,124]. For PPARs, this raises an important question: how much of PPAR biology is determined by canonical PPAR-RXR complexes, and how much may be influenced by alternative dimerization, RXR competition, or cross-talk with other nuclear receptors?

This direction is worth pursuing because RXR is a common heterodimeric partner for many nuclear receptors, including PPARs, LXRs, FXR, RARs, TRs, VDR, CAR, PXR, and others [42,123,124,125]. Therefore, changes in RXR availability or ligand occupancy can influence multiple transcriptional programs simultaneously [124,125]. In addition, RXR homodimers can bind functional PPREs and activate PPAR target genes in vivo, suggesting that PPRE regulation may not always require a PPAR-containing complex [126]. Furthermore, nuclear receptor cross-talk involving PPARs, LXRs, CAR, and other RXR partners is relevant to lipid metabolism, inflammation, obesity, atherosclerosis, hepatic metabolism, and xenobiotic responses [127,128]. These pathways are not independent, and it is worth noting that they converge on metabolic and inflammatory gene programs that are central to many PPAR-linked diseases.

The possibility of noncanonical PPAR-containing dimers should therefore be considered within the broader framework of nuclear receptor crosstalk rather than only as stable PPAR homodimerization. Nuclear receptor crosstalk can arise from overlapping signaling pathways, competition for shared partners such as RXR, or direct physical association between receptors. Importantly, De Bosscher et al. proposed that, in addition to well-characterized RXR heterodimers such as PPAR-RXR, more transient heterodimers may form between nuclear receptors, including examples such as GR-PPAR or PPAR-ERR complexes [129]. These assemblies may be context-dependent, ligand-dependent, and cell-type-specific, and they may not require both partners to bind DNA in a canonical manner [129]. This concept is particularly relevant for PPAR biology because PPARs sit at the intersection of lipid metabolism, inflammation, and endocrine signaling. For example, Bougarne et al. showed that co-activation of PPARα and GRα produces cooperative anti-inflammatory effects by attenuating NF-κB-driven transcription, reducing MSK1 phosphorylation, and promoting additive repression of inflammatory genes in epithelial cells and hepatocytes [130].

Moreover, interactions between PPARγ and other nuclear receptors have been proposed in specific biological contexts. For example, the photoreceptor-specific nuclear receptor PNR was reported to interact with PPARγ in retinal tissue, suggesting that PPARγ may participate in noncanonical nuclear receptor complexes outside the classical metabolic paradigm [131]. Although such examples remain limited, they support a broader future direction: PPAR dimerization should be studied not only as a fixed PPAR-RXR event but as part of a dynamic nuclear receptor interaction network.

From an MD perspective, this area is largely unexplored. Future studies could compare canonical PPAR-RXR complexes with RXR homodimers bound to PPREs, investigate competition between RXR partners, and model PPARs with noncanonical nuclear receptor partners where experimental evidence exists. Such work would connect structural dynamics to pathway-level biology, especially in diseases where lipid metabolism, inflammation, adipogenesis, retinoid signaling, and xenobiotic responses intersect.

### 5.6. PPARγ, Mesenchymal Stem-Cell Differentiation, and Transcription-Factor Crosstalk

A further direction in which PPAR MD could expand is cell-fate regulation. PPARγ is a central regulator of adipogenic differentiation, while osteogenic differentiation depends on transcriptional networks involving RUNX2, SOX9, Wnt signaling, BMP signaling, and glucocorticoid receptor activity [132,133,134,135]. Because adipogenic and osteogenic fates of mesenchymal stromal/stem cells are often inversely related, PPARγ represents a key structural and regulatory link between receptor pharmacology and differentiation biology. This context also provides a bridge to MD studies that are not directly focused on PPARγ but address transcription-factor interactions relevant to mesenchymal differentiation. For example, our previous MD study investigated how dexamethasone influences the interaction between the glucocorticoid receptor and SOX9 at atomistic resolution [136]. Although this system did not include PPARγ directly, it is conceptually relevant because dexamethasone-dependent mesenchymal stromal-cell differentiation has been linked to changes in SOX9 and PPARγ expression, and Della Bella et al. showed that dexamethasone promotes osteogenic differentiation by inhibiting SOX9 rather than directly increasing RUNX2, while also affecting PPARγ-associated adipogenic features [137].

This connection suggests that future PPARγ simulations should not be limited to ligand pharmacology but should also consider transcriptional crosstalk in cell differentiation. For instance, MD could be used to study how PPARγ ligands alter co-regulator recruitment in adipogenic versus osteogenic contexts, or how PPARγ-containing transcriptional complexes are affected by glucocorticoid signaling. Simulations could also be integrated with transcriptomic data from mesenchymal differentiation models to identify which structural states are most relevant to cell-fate decisions. This would extend PPAR MD from receptor pharmacology toward systems-level differentiation biology.

## 6. Conclusions

MD has substantially reshaped the structural interpretation of PPAR function. The classical model in which agonists activate PPARs mainly by stabilizing H12 remains useful but is no longer sufficient. Current evidence supports an ensemble-based mechanism in which PPAR activity emerges from ligand-dependent redistribution of conformational populations involving H12, AF-2, H3, the β-sheet region, Ω-loop, ligand-entry pathways, co-regulator surfaces, RXR interfaces, and DNA-bound transcriptional assemblies.

Among the three isoforms, PPARγ is the clear benchmark for dynamic and integrative structural studies. Its conformational landscape has been explored through classical and enhanced MD, NMR, HDX-MS, crystallography, mutagenesis, and co-regulator assays. Across ligand classes, the literature points to distinct receptor substates stabilized by full agonists, partial agonists, antagonists, inverse agonists, endogenous lipids, and covalent or non-covalent modulators, rather than to different static poses alone. In contrast, PPARα and PPARβ/δ remain less comprehensively characterized, especially in apo, antagonist-bound, mutant, PTM-modified, and full heterodimeric contexts. This imbalance is a major opportunity for future work.

## Data Availability

No new data were created or analyzed in this study. Data sharing is not applicable to this article.

## Supplementary Materials

The following supporting information can be downloaded at: https://www.mdpi.com/article/10.3390/cells15151395/s1, Table S1: Summary of key studies supporting Section 1—conformational dynamics of PPAR nuclear receptors. Table S2: Summary of MD-focused and integrative studies on PPARs-protein interactions. Table S3: Summary studies on PPARs disease-associated mutations and structural/dynamic mechanisms. Table S4: Summary of MD-focused studies on PPAR-ligand interactions.
