# Supplementary material for: Molecular Dynamics of PPAR Nuclear Receptors: From Ligand Binding to Transcriptional Regulation
Source: Cells. 2026 Jul 31;15(15):1395. doi: 10.3390/cells15151395 (PMC13464093; doi:10.3390/cells15151395)
Supplement: Supplementary file 1 [file cells-15-01395-s001.zip › cells-4452222-supplementary.pdf]

Supporting Information

Molecular Dynamics of PPAR Nuclear Receptors: From Ligand Binding to Transcriptional Regulation

Filip Stojceski[1] and Andrea Danani[1]*

[1]SUPSI, IDSIA - Dalle Molle Institute for Artificial Intelligence (USI-SUPSI), Polo universitario Lugano - Campus Est, Via la Santa 1, CH-6962, Lugano, Switzerland.

**Corresponding author: Filip Stojceski; filip.stojceski@supsi.ch*

Summary tables

Table S1: Summary of key studies supporting Section 1 - conformational dynamics of PPAR nuclear receptors.

Ref.	***Study***	***Isoform/ system***	***Main focus***	***MD / computational role***	***Other techniques***	***Main analyses or structural elements***
[1]	Nolte et al., 1998	PPARγ LBD	Foundational PPARγ LBD structure	None	X-ray crystallography	Ligand-binding pocket, AF-2 surface, H12, coactivator assembly
[2]	Bruning et al., 2007	PPARγ LBD	Partial agonism and H12-independent activation	None or limited computational support	X-ray crystallography, HDX-MS	H12, β-sheet region, H3, partial agonist binding
[3]	Kallenberger et al., 2003	Nuclear receptor LBDs; relevant to PPARγ dynamics	Dynamic mechanism of nuclear receptor activation	None	Biophysical dynamics assays	H12 mobility
[4]	Kojetin & Burris, 2013	Nuclear receptors, including PPARγ	Conceptual framework for ligand-regulated dynamics	Review of computational and experimental dynamics	NMR, HDX-MS, crystallography literature	LBD conformational ensembles, ligand efficacy
[5]	Fratev, 2016	PPARγ LBD	H12 conformational landscape	Accelerated MD, metadynamics, conventional MD	None	H12, agonist-like and antagonist-like conformations
[6]	Batista et al., 2015	PPARγ LBD	H12 conformational diversity	Computational conformational analysis / MD-based modeling	None	H12 substates

[7]	Chrisman et al., 2018	PPARγ LBD	Conformational ensemble directing activation	Molecular simulations integrated with experiments	NMR, HDX-MS, crystallography, co-regulator assays	AF-2 surface, H12, ensemble populations
[8]	Heidari et al., 2019	PPARγ LBD	Repressive conformational states	Accelerated MD	NMR, HDX-MS, mutagenesis, crystallography	H3 charge clamp, H12, Ω-loop, salt-bridge network
[9]	Falbo et al., 2025	PPARγ LBD	Apo-to-ligand-bound conformational shifts	Classical MD, Gaussian accelerated MD, machine-learning clustering, WT-MetaD	None	H12 transition, free-energy landscape, apo/agonist/antagonist-like states
[10]	Zhao et al., 2026	PPARγ LBD	Allosteric landscape and conformational-state transitions	Extensive MD, Markov-state modeling, and energetic analysis	None	Ligand-selectable conformational states, allosteric transitions, H12/AF-2-linked dynamics
[11]	Basith et al., 2019	PPARα LBD	Intramolecular signal transduction	Unbiased MD of apo, agonist-bound, and antagonist-bound PPARα	None	RMSD/RMSF, hydrogen bonds, PCA, DCCM, residue-network analysis
[12]	Chandra et al., 2008	Intact PPARγ-RXRα complex on DNA	Multidomain receptor architecture	None	X-ray crystallography	PPARγ-RXRα heterodimer, DNA, ligands, coactivator peptides
[13]	Kojetin et al., 2015	RXR heterodimers including PPARγ-RXRα	Signal transduction in RXR heterodimers	None or supporting structural interpretation	NMR, crystallography, HDX-MS	Heterodimer interface, allosteric cooperation
[14]	Ricci et al., 2016	PPARγ-RXRα complex	Allosteric pathways in the heterodimer	Molecular dynamics and statistical network analysis	None	Ω-loops, DNA-binding domain, correlated motions, allosteric pathways
[15]	Xu et al., 2001	PPARα, PPARβ/δ, PPARγ LBDs	Isoform ligand-selectivity determinants	Structural comparison; computationally useful	X-ray crystallography	Ligand-binding pocket, subtype-selective residues, AF-2 helix
[16]	Cronet et al., 2001	PPARα and PPARγ LBDs	Dual agonist binding and activation	None	X-ray crystallography	AZ242 binding, conserved activation interactions, H12
[17]	Fyffe et al., 2006	PPARβ/δ LBD	Endogenous fatty-acid stabilization	None	X-ray crystallography	Endogenous fatty acids, activated LBD conformation
[18]	Batista et al., 2012	PPARβ/δ LBD	PPARβ/δ-selective ligand binding	Comparative structural/computational interpretation	X-ray crystallography	GW0742 binding, Val312, Ile328, subtype-selective pocket features
[19]	Shang et al., 2020	PPARγ LBD	Molecular switch for repression and activation	Structural/dynamic interpretation	Crystallography, HDX-MS, corepressor/coactivator assays	Inverse agonism, corepressor recruitment, repressive conformations
[20]	Capelli et al., 2016	PPAR LBDs	Partial vs full activation by ligand binding mode	Structural/computational interpretation	X-ray crystallography, biochemical assays	Ligand binding mode, partial/full activation, H12/AF-2
[21]	Shang et al., 2018	PPARγ LBD	Cooperative ligand co-binding	MD-supported structural interpretation	X-ray crystallography, NMR, mutagenesis, biochemical/cellular assays	Orthosteric pocket, alternate ligand site, Ω-loop, fatty acids

[22]	Agosta & Cozzini, 2024	PPARγ with rosiglitazone and oleic acid	Ω-loop contribution to ligand-mediated activation	MD combined with HINT hydropathic-interaction analysis	Computational energetic interpretation	Ω-loop, H3, cooperative ligand effects, intramolecular interaction patterns
[23]	Shang & Kojetin, 2021	PPARγ LBD	Ligand-binding mechanism	Computational and structural interpretation	Structural biology, ligand-binding assays	Encounter complex, induced fit, orthosteric transition
[24]	Sullivan et al., 2020	PPARα, PPARβ/δ, PPARγ	Pan-agonist chiglitazar comparison	Docking + 3 μs MD for each receptor-ligand complex; MM-GBSA	None	H12, residue interactions, ligand flexibility, binding energy

Table S2: Summary of MD-focused and integrative studies on PPARs-protein interactions.

Ref.	***Study***	***PPAR system***	***Interaction type***	***MD/computational role***	***Other techniques***	***Main structural elements***
[25]	Venäläinen et al., 2010	PPARα-RXRα heterodimer	RXR heterodimerization; inter-receptor allostery	Molecular dynamics simulations of heterodimeric receptor states	Structural modeling	PPARα LBD, RXRα LBD, heterodimer interface, AF-2 region
[26]	Amber-Vitos et al., 2016	PPAR-RXR complex	Regulatory-molecule effects on heterodimer architecture	All-atom MD simulations of the PPAR-RXR complex	Structural modeling	Heterodimer orientation, receptor-receptor contacts, ligand-dependent interdomain communication
[27]	de Vera et al., 2017	PPARγ-RXRα with ligand, DNA, and co-regulator context	DNA-dependent regulation of co-regulator/nuclear receptor interaction	Not primarily MD; important integrative structural/biophysical reference for interpreting MD of transcription complexes	Structural biology, ligand/co-regulator binding assays	PPARγ-RXRα, DNA response element, co-regulator interface, AF-2 surface
[28]	Azam & Bello, 2022	RXRα-PPARγ versus RXRα-PPARγ-DNA	RXR heterodimerization and DNA-bound transcription complex	Microsecond-scale MD simulations; MM-GBSA; PCA; free-energy landscape analysis; dynamic cross-correlation / energetic comparison	Structural modeling from available complexes	RXRα-PPARγ interface, DNA-bound and DNA-free states, LBD/DBD organization
[29]	Lemkul et al., 2015	PPARγ-RXRα-DNA complex	Phosphorylation-dependent regulation of transcription complex dynamics	Atomistic MD simulations of phosphorylated and unphosphorylated PPARγ-RXRα-DNA complexes; collective-motion analysis	Structural modeling	S245/S273 phosphorylation region, LBD-LBD interface, AF-2 region, DNA-bound receptor complex
[30]	Zheng et al., 2018	PPARγ LBD with ligands and regulatory peptides	Coactivator/corepressor-linked H12 conformational landscape	Not primarily MD; provides experimental conformational constraints for interpreting MD	Chemical crosslinking MS, structural/biochemica l assays	H12 / activation helix, ligand-dependent antagonist states, peptide-regulated conformations
[31]	Frkic et al., 2023	PPARγ LBD with ligand and corepressor context	Corepressor binding; inverse agonism; H12 ensemble expansion	Not primarily MD; ensemble-based structural interpretation relevant to MD	Structural biology, mass spectrometry, biochemical assays	H12 ensemble, corepressor-binding surface, alternate ligand conformations

[32]	Mottin et al., 2015	PPARγ and CDK5/p25 kinase complex	Kinase recognition; phosphorylation-site accessibility	Protein-protein docking plus adaptive biasing force simulations	Structural modeling	CDK5/p25 interface, PPARγ β-sheet region, Ω-loop, S273/S245 phosphorylation region
[33]	Montanari et al., 2020	PPARγ phosphorylation site and ligand-regulated inhibition	Kinase-linked phosphorylation inhibition	In silico modeling integrated with solution-based mechanistic experiments	Native MS / solution biophysics, biochemical assays, structural modeling	S273/S245 region, β-sheet, ligand pocket, phosphorylation-regulatory surface
[34]	Malospirito et al., 2025	Acetylated PPARγ with SIRT1	SIRT1 recognition of acetylated PPARγ	Molecular modeling / MD-based interface analysis of PPARγ-SIRT1 anchoring	None	K268/K293 acetylation, SIRT1 N-terminal domain, deacetylase-recognition interface
[35]	Malospirito et al., 2026	PPARγ with S273 phosphorylation and K268/K293 acetylation	Phosphorylation-acetylation crosstalk	Computational modeling of PTM-dependent PPARγ regulation	CDK5/SIRT1 binding and transcriptional analyses	S273 phosphorylation, K268/K293 acetylation, CDK5/SIRT1 regulatory interface
[36]	Biswas et al., 2026	PPARα / BPA / ubiquitination context	Ligand-linked ubiquitination regulation	MD of BPA binding to PPARα integrated with ubiquitinome/network analysis; not a covalently ubiquitinated PPARα MD model	Ubiquitinome analysis, ligase/deubiquitinase analysis, trophoblast-cell assays	PPARα ligand-binding region, lysine-rich flexible region, ubiquitination machinery

Table S3: Summary studies on PPARs disease-associated mutations and structural/dynamic mechanisms.

Ref.	***Study***	***PPAR system / variant(s)***	***Disease context***	***MD / computational role***	***Other techniques***	***Main structural elements***
[37]	Agostini et al., 2006	PPARγ mutants in DBD and LBD; includes V290M, R425C, P467L	Familial partial lipodystrophy, severe insulin resistance	No MD; foundational functional/genetic reference	Transactivation assays, DNA-binding assays, coactivator interaction assays, nuclear localization	DBD, LBD, dominant-negative receptor behavior
[38]	Monajemi et al., 2007	PPARγ2 R194W	Familial partial lipodystrophy type 3	No MD; structural/functional disease reference	Genetic analysis, functional assays, DNA-binding analysis	DNA-binding domain, PPRE recognition interface
[39]	Lori et al., 2014	PPARγ F360L / PPARγ2 F388L	Familial partial lipodystrophy	MD-supported structural interpretation	X-ray crystallography, spectroscopy, calorimetry, transactivation assays	LBD stability, ligand-binding pocket, AF-2/H12-related response
[40]	Petrosino et al., 2017	Multiple PPARγ LBD SNPs, including V290M, R357A, F360L, P467L, Q286P, R288H, E324K, E460K	Cancer, lipodystrophy, insulin resistance, metabolic disease	Molecular dynamics used to evaluate structural/dynamic consequences of selected variants	Biochemical assays, spectroscopy, structural modeling	LBD stability, ligand responsiveness, local flexibility, AF-2-linked dynamics
[41]	Stalin et al., 2022	PPARγ SNPs, including C162S, R166W, Q286P, Q314P, P467L	Obesity, diabetes, cancer	In silico SNP prioritization plus MD simulations; RMSD, RMSF, radius of gyration and stability analyses	Sequence/structure prediction tools, pathogenicity prediction	Global receptor stability, local residue flexibility, compactness

[42]	Taghvaei & Saremi, 2022	PPARγ2 P12A	Type 2 diabetes, cardiovascular disease, NAFLD	Homology/modeling, GROMACS MD, essential dynamics, RMSD/RMSF/Rg/SASA/H-bond analyses	Stability-prediction tools, structure validation	N-terminal PPARγ2 region, global flexibility, essential motions
[43]	Madsen et al., 2022	PPARγ R212Q and E379K	Familial partial lipodystrophy, impaired adipogenesis	Structural modeling / interface interpretation; not primarily MD	Genome-wide enhancer analyses, biochemical assays, adipogenesis assays, structural analysis	PPARγ-DNA interface, PPARγ-RXRα interface, enhancer activation complex
[44]	Ruscica et al., 2019	PPARα polymorphisms, including L162V and V227A	Dyslipidemia, metabolic disorders, atherosclerosis	No MD; review-level disease genetics reference	Literature synthesis of genetic, metabolic and cardiovascular studies	PPARα sequence variants, lipid-metabolism regulation
[45]	Jeninga et al., 2007	PPARγ R425C	Familial partial lipodystrophy	MD-supported interpretation of altered salt-bridge behavior	Functional assays, structural modeling	Conserved salt-bridge network, LBD stability, receptor function

Table S4: Summary of MD-focused studies on PPAR-ligand interactions.

Ref.	***Study***	***PPAR / ligand class***	***Interaction type***	***MD / computational approach***	***Other techniques***	***Main structural elements***
[46]	Genest et al., 2008	PPARγ LBD with bound ligand	Ligand dissociation / escape pathways	Molecular dynamics simulations probing ligand-escape routes	Structural modeling	Ligand-binding pocket, transient exit channels, helices and loops surrounding the pocket
[47]	Aci-Sèche et al., 2011	PPARγ LBD with GW0072	Ligand entry pathways	Targeted molecular dynamics of ligand entry into the LBD	Structural modeling	Entry channels, ligand-binding pocket, hydrophobic guiding interactions
[48]	Liberato et al., 2012	PPARγ and pan-PPAR fatty-acid ligands	Endogenous-like lipid activation / partial agonism	MD-supported structural interpretation	X-ray crystallography, B-factor analysis, transactivation assays	H12, AF-2 surface, fatty-acid chain-length-dependent pocket interactions
[49]	Hughes et al., 2014	PPARγ LBD with alternate-site ligands	Alternate ligand binding / noncanonical modulation	Structural and dynamic interpretation relevant to MD	X-ray crystallography, NMR/biophysical assays	Alternate binding site near Ω-loop, orthosteric pocket, H3/β-sheet region
[50]	Kamata et al., 2020	PPARα LBD with endogenous fatty acids and fibrates	Fatty-acid and fibrate recognition	MD integrated with structural interpretation	X-ray crystallography, mutagenesis, functional assays	PPARα ligand-binding pocket, fatty-acid binding modes, H12/AF-2 region
[51]	Liu et al., 2018	PPARα/γ with ASN15761007-like candidates	Dual agonist discovery	Virtual screening, ADMET prediction, MD simulation	In silico pharmacokinetic/toxicity assessment	PPARα and PPARγ active sites, ligand stability, agonist-like binding conformations
[52]	D'Aniello et al., 2019	PPARα/γ with phytocannabinoids	Natural dual agonists	Computational binding prediction with MD-relevant structural interpretation	Luciferase reporter assays, adipocyte/hepatocyte functional assays	PPARα and PPARγ ligand-binding pockets

[53]	Iannotti et al., 2020	PPARγ with cannabimovone	Cannabis-derived PPARγ agonist	Docking and 50 ns MD simulations	Functional reporter assays	PPARγ ligand-binding pocket, agonist-interaction network
[54]	Kumar et al., 2022	PPARγ with novel glitazones	Synthetic agonist design	Docking and MD simulation integrated with ligand optimization	Synthesis, competitive binding, transcription assays	PPARγ ligand-binding pocket, agonist-like interaction stability
[55]	Kumar et al., 2023	PPARγ with imidazolidinedione / thioxothiazolidinone glitazones	Central PPARγ agonism	Computational modeling and MD simulation	Synthesis, in vitro binding, transcription analysis	Ligand-binding pocket, agonist-like binding mode, receptor stability
[56]	Haider et al., 2024	PPARγ agonist candidates	ML-assisted agonist discovery	ML filtering, docking, MD simulation, binding-energy analysis	In silico screening	PPARγ active site, ligand stability, residue-level interactions
[57]	She et al., 2026	PPARγ with pemetrexed	Partial agonism	Microsecond-scale MD, PCA, free-energy landscape, correlated-motion analysis	Computational structural analysis	H12, AF-2, partial-agonist binding mode, correlated motions
[58]	Lokhande et al., 2022	PPARγ with kaempferol, quercetin, and resveratrol derivatives	Natural-product agonist screening	Docking, MD, MM/GBSA	None	PPARγ ligand-binding pocket, interaction persistence, binding stability
[59]	Prabitha et al., 2022	PPARγ with rosiglitazone-bound conformational ensemble	MD-derived pharmacophore modeling	MD trajectories used to generate multi-conformational pharmacophore models	Virtual screening / pharmacophore modeling	Multiple receptor conformations, ligand-pocket flexibility
[60]	Perricone et al., 2017	PPARα	MD-shared pharmacophore development	MD-derived representative frames used for pharmacophore screening	Virtual screening	PPARα ligand-binding pocket, receptor flexibility, early-enrichment screening
[61]	Ahmed et al., 2021	PPARγ agonist candidates	Antidiabetic agonist design	Virtual screening, MD, DFT, QSAR	Computational filtering	PPARγ ligand-binding pocket, predicted agonist stability
[62]	Mandal et al., 2025	PPARβ/δ agonist candidates	Drug-repurposing / anti-steatotic ligand discovery	HTVS, MD, pharmacokinetics analysis	In vitro anti-steatotic validation	PPARβ/δ ligand-binding pocket, agonist-like stability
[63]	Chen et al., 2012	PPARα, PPARβ/δ, and PPARγ with TCM-derived ligands	Pan-PPAR agonist discovery	Structure-based pharmacophore, docking, and MD-based refinement	In silico screening	Three PPAR ligand-binding pockets, pan-agonist interaction patterns
[64]	Mandal et al., 2024	PPARα, PPARβ/δ, and PPARγ with pharmacological ligands	Pan-PPAR agonist screening	HTVS, docking, and molecular docking/dynamics analysis	ADMET / pharmacological filtering	Isoform-specific ligand pockets, pan-PPAR binding stability
[65]	Mandal et al., 2025	PPARα, PPARβ/δ, and PPARγ with polydatin	Pan-PPAR agonism / anti-NAFLD mechanism	HTVS, docking/MD analysis	In vitro HepG2 steatosis model	Pan-PPAR ligand recognition, lipid-metabolism-related activation
[66]	Feng et al., 2019	PPARα/γ with CHEMBL230490-like candidates	Dual agonist discovery	Pharmacophore screening, docking, ADMET, MD simulation	In silico filtering	PPARα and PPARγ pockets, dual-agonist stability
[67]	Nath et al., 2020	PPARα/γ dual agonists	Metabolic-disease dual agonism	Structure-based docking and MD simulations	None	PPARα/γ active sites, stable dual-receptor interaction patterns

[68]	Chen & Chen, 2014	PPARγ with traditional Chinese medicine compounds	Natural-product agonist screening	Bioactivity prediction, virtual screening, and MD simulation	In silico screening	PPARγ ligand-binding pocket, predicted agonist interactions
[69]	Gangadhariah et al., 2023	PPARγ with eriocitrin and metabolites	Citrus nutraceutical partial agonism	Docking and MD simulation	None	PPARγ pocket, partial-agonist-like interaction stability
[70]	Wong et al., 2023	PPARγ with Nelumbinis Folium compounds	Weight-management ligand screening	Docking and MD simulations	Network/phytochemical analysis	PPARγ ligand-binding pocket, natural-compound stability
[71]	Singh et al., 2026	PPARγ with Moringa oleifera phytochemicals	Nutraceutical agonist screening	In silico screening and MD analysis	ADMET / computational filtering	PPARγ receptor pocket, phytochemical binding stability
[72]	Bhongade et al., 2025	PPARγ with natural compounds	Inflammatory-bowel-disease ligand screening	Docking and MD simulations	In silico ligand prioritization	PPARγ ligand-binding pocket, stable modulator interactions
[73]	Zhao et al., 2026	PPARγ with neferine	Disease-oriented natural-product binding	Docking and MD suggesting stable PPARγ binding	Animal experiments, antagonist validation	PPARγ binding pocket, anti-inflammatory pathway context
[74]	Ballav et al., 2023	PPARα with polyphenolic derivatives	Natural-product-inspired PPARα candidates	Docking, MD, MM/GBSA	None	PPARα LBD, kaempferol/resveratrol/quercetin derivative interactions
[75]	Ayenero & Oduola, 2026	PPARα with Solanum erianthum phytochemicals	Oxidative-stress liver-injury ligand screening	Docking, MD, MM/GBSA	In silico multitarget analysis	PPARα LBD, apigenin-like binding stability
[76]	Muralikumar et al., 2017	PPARγ LBD with bioactive lipids	Lipid-PPARγ binding	Docking, MD simulations, binding free-energy calculations	None	Ligand-binding pocket, residue-level lipid interactions
[77]	Lian et al., 2024	PPARγ with natural-product partial agonists	Partial agonist discovery	Virtual screening, MD simulation, MM-PBSA analysis	In silico filtering	PPARγ pocket regions outside direct AF-2 interaction; H12-independent stabilization
[78]	Kuo et al., 2025	PPARγ with non-covalent inverse agonists	Inverse agonism / repression	Structural analysis and MD simulations	Functional assays	H12, repressive conformations, ligand-binding pocket
[79]	Padhy et al., 2024	PPARγ inhibitors	PPARγ inhibition / anti-obesity strategy	QSAR, docking, MD simulation	Synthesis and biological evaluation	PPARγ ligand-binding pocket, inhibitor interaction stability
[80]	Wang et al., 2025	PPARγ with partial agonist TWSZ-5	Novel partial-agonist pocket	MD simulations of ligand–receptor stability	Docking / structure-based discovery	Newly proposed binding pocket, partial-agonist interaction network
[81]	Liu et al., 2026	PPARγ partial agonist candidates	Type 2 diabetes partial-agonist discovery	FMO calculations, ML, docking, interaction fingerprints, MD	In silico prioritization	PPARγ pocket, partial-agonist interaction patterns
[82]	Kaya et al., 2024	PPARγ and α-glucosidase dual-target compounds	Multitarget antidiabetic discovery	Docking, MD simulations, MM/PBSA	In silico dual-target analysis	PPARγ pocket, α-glucosidase pocket, multitarget ligand stability
[83]	Kagimbo et al., 2026	PPARγ / multitarget antidiabetic candidates	Multitarget plant-compound screening	In silico multitarget workflow with MD	In silico target screening	PPARγ and additional antidiabetic targets
[84]	Jain et al., 2026	PPARγ with TZD-oxazole derivatives	Synthetic agonist design	3D-QSAR, virtual screening, MD simulations	In silico optimization	PPARγ ligand-binding pocket, agonist-like stability

Bibliography

1. Nolte, R.T.; Wisely, G.B.; Westin, S.; Cobb, J.E.; Lambert, M.H.; Kurokawa, R.; Rosenfeld, M.G.; Willson, T.M.; Glass, C.K.; Milburn, M.V. Ligand Binding and Co-Activator Assembly of the Peroxisome Proliferator-Activated Receptor-γ. *Nature* **1998**, *395*, 137–143, doi:10.1038/25931.

2. Bruning, J.B.; Chalmers, M.J.; Prasad, S.; Busby, S.A.; Kamenecka, T.M.; He, Y.; Nettles, K.W.; Griffin, P.R. Partial Agonists Activate PPARγ Using a Helix 12 Independent Mechanism. *Structure* **2007**, *15*, 1258–1271, doi:10.1016/j.str.2007.07.014.

3. Kallenberger, B.C.; Love, J.D.; Chatterjee, V.K.K.; Schwabe, J.W.R. A Dynamic Mechanism of Nuclear Receptor Activation and Its Perturbation in a Human Disease. *Nat Struct Biol* **2003**, *10*, 136–140, doi:10.1038/nsb892.

4. Kojetin, D.J.; Burris, T.P. Small Molecule Modulation of Nuclear Receptor Conformational Dynamics: Implications for Function and Drug Discovery. *Molecular Pharmacology* **2013**, *83*, 1–8, doi:10.1124/mol.112.079285.

5. Fratev, F. PPARγ Helix 12 Exhibits an Antagonist Conformation. *Phys. Chem. Chem. Phys.* **2016**, *18*, 9272–9280, doi:10.1039/C5CP06729D.

6. Batista, M.R.B.; Martínez, L. Conformational Diversity of the Helix 12 of the Ligand Binding Domain of PPARγ and Functional Implications. *J. Phys. Chem. B* **2015**, *119*, 15418–15429, doi:10.1021/acs.jpcb.5b09824.

7. Chrisman, I.M.; Nemetchek, M.D.; De Vera, I.M.S.; Shang, J.; Heidari, Z.; Long, Y.; Reyes-Caballero, H.; Galindo-Murillo, R.; Cheatham, T.E.; Blayo, A.-L.; et al. Defining a Conformational Ensemble That Directs Activation of PPARγ. *Nat Commun* **2018**, *9*, 1794, doi:10.1038/s41467-018-04176-x.

8. Heidari, Z.; Chrisman, I.M.; Nemetchek, M.D.; Novick, S.J.; Blayo, A.-L.; Patton, T.; Mendes, D.E.; Diaz, P.; Kamenecka, T.M.; Griffin, P.R.; et al. Definition of Functionally and Structurally Distinct Repressive States in the Nuclear Receptor PPARγ. *Nat Commun* **2019**, *10*, 5825, doi:10.1038/s41467-019-13768-0.

9. Falbo, E.; Delre, P.; Lavecchia, A. From Apo to Ligand-Bound: Unraveling PPARγ-LBD Conformational Shifts via Advanced Molecular Dynamics. *ACS Omega* **2025**, *10*, 13303–13318, doi:10.1021/acsomega.4c11128.

10. Zhao, J.; Yang, Y.; Zhang, Z.; Zhang, L.; Zhang, X.; Yang, Z. Mapping the Allosteric Landscape of PPAR$_{\gamma}$: A Markov State Modeling and Energetic Analysis Approach. *J. Chem. Inf. Model.* **2026**, *66*, 1190–1202, doi:10.1021/acs.jcim.5c02527.

11. Basith, S.; Manavalan, B.; Shin, T.H.; Lee, G. A Molecular Dynamics Approach to Explore the Intramolecular Signal Transduction of PPAR-α. *IJMS* **2019**, *20*, 1666, doi:10.3390/ijms20071666.

12. Chandra, V.; Huang, P.; Hamuro, Y.; Raghuram, S.; Wang, Y.; Burris, T.P.; Rastinejad, F. Structure of the Intact PPAR-γ–RXR-α Nuclear Receptor Complex on DNA. *Nature* **2008**, *456*, 350–356, doi:10.1038/nature07413.

13. Kojetin, D.J.; Matta-Camacho, E.; Hughes, T.S.; Srinivasan, S.; Nwachukwu, J.C.; Cavett, V.; Nowak, J.; Chalmers, M.J.; Marciano, D.P.; Kamenecka, T.M.; et al. Structural Mechanism for Signal Transduction in RXR Nuclear Receptor Heterodimers. *Nat Commun* **2015**, *6*, 8013, doi:10.1038/ncomms9013.

14. Ricci, C.G.; Silveira, R.L.; Rivalta, I.; Batista, V.S.; Skaf, M.S. Allosteric Pathways in the PPARγ-RXRα Nuclear Receptor Complex. *Sci Rep* **2016**, *6*, 19940, doi:10.1038/srep19940.

15. Xu, H.E.; Lambert, M.H.; Montana, V.G.; Plunket, K.D.; Moore, L.B.; Collins, J.L.; Oplinger, J.A.; Kliewer, S.A.; Gampe, R.T.; McKee, D.D.; et al. Structural Determinants of Ligand Binding Selectivity between the Peroxisome Proliferator-Activated Receptors. *Proc. Natl. Acad. Sci. U.S.A.* **2001**, *98*, 13919–13924, doi:10.1073/pnas.241410198.

16. Cronet, P.; Petersen, J.F.W.; Folmer, R.; Blomberg, N.; Sjöblom, K.; Karlsson, U.; Lindstedt, E.-L.; Bamberg, K. Structure of the PPARα and -γ Ligand Binding Domain in Complex with AZ 242; Ligand Selectivity and Agonist Activation in the PPAR Family. *Structure* **2001**, *9*, 699–706, doi:10.1016/S0969-2126(01)00634-7.

17. Fyffe, S.A.; Alphey, M.S.; Buetow, L.; Smith, T.K.; Ferguson, M.A.J.; Sørensen, M.D.; Björkling, F.; Hunter, W.N. Recombinant Human PPAR-β/δ Ligand-Binding Domain Is Locked in an Activated Conformation by Endogenous Fatty Acids. *Journal of Molecular Biology* **2006**, *356*, 1005–1013, doi:10.1016/j.jmb.2005.12.047.

18. Batista, F.A.H.; Trivella, D.B.B.; Bernardes, A.; Gratieri, J.; Oliveira, P.S.L.; Figueira, A.C.M.; Webb, P.; Polikarpov, I. Structural Insights into Human Peroxisome Proliferator Activated Receptor Delta (PPAR-Delta) Selective Ligand Binding. *PLoS ONE* **2012**, *7*, e33643, doi:10.1371/journal.pone.0033643.

19. Shang, J.; Mosure, S.A.; Zheng, J.; Brust, R.; Bass, J.; Nichols, A.; Solt, L.A.; Griffin, P.R.; Kojetin, D.J. A Molecular Switch Regulating Transcriptional Repression and Activation of PPARγ. *Nat Commun* **2020**, *11*, 956, doi:10.1038/s41467-020-14750-x.

20. Capelli, D.; Cerchia, C.; Montanari, R.; Loiodice, F.; Tortorella, P.; Laghezza, A.; Cervoni, L.; Pochetti, G.; Lavecchia, A. Structural Basis for PPAR Partial or Full Activation Revealed by a Novel Ligand Binding Mode. *Sci Rep* **2016**, *6*, 34792, doi:10.1038/srep34792.

21. Shang, J.; Brust, R.; Mosure, S.A.; Bass, J.; Munoz-Tello, P.; Lin, H.; Hughes, T.S.; Tang, M.; Ge, Q.; Kamenekca, T.M.; et al. Cooperative Cobinding of Synthetic and Natural Ligands to the Nuclear Receptor PPARγ. *eLife* **2018**, *7*, e43320, doi:10.7554/eLife.43320.

22. Agosta, F.; Cozzini, P. A Combined Molecular Dynamics and Hydropathic INTeraction (HINT) Approach to Investigate Protein Flexibility: The PPARγ Case Study. *Molecules* **2024**, *29*, 2234, doi:10.3390/molecules29102234.

23. Shang, J.; Kojetin, D.J. Structural Mechanism Underlying Ligand Binding and Activation of PPARγ. *Structure* **2021**, *29*, 940-950.e4, doi:10.1016/j.str.2021.02.006.

24. Sullivan, H.-J.; Wang, X.; Nogle, S.; Liao, S.; Wu, C. To Probe Full and Partial Activation of Human Peroxisome Proliferator-Activated Receptors by Pan-Agonist Chiglitazar Using Molecular Dynamics Simulations. *PPAR Research* **2020**, *2020*, 1–24, doi:10.1155/2020/5314187.

25. Venäläinen, T.; Molnár, F.; Oostenbrink, C.; Carlberg, C.; Peräkylä, M. Molecular Mechanism of Allosteric Communication in the Human PPARα-RXRα Heterodimer. *Proteins* **2010**, *78*, 873–887, doi:10.1002/prot.22613.

26. Amber-Vitos, O.; Chaturvedi, N.; Nachliel, E.; Gutman, M.; Tsfadia, Y. The Effect of Regulating Molecules on the Structure of the PPAR-RXR Complex. *Biochimica et Biophysica Acta (BBA) - Molecular and Cell Biology of Lipids* **2016**, *1861*, 1852–1863, doi:10.1016/j.bbalip.2016.09.003.

27. De Vera, I.M.S.; Zheng, J.; Novick, S.; Shang, J.; Hughes, T.S.; Brust, R.; Munoz-Tello, P.; Gardner, W.J.; Marciano, D.P.; Kong, X.; et al. Synergistic Regulation of Coregulator/Nuclear Receptor Interaction by Ligand and DNA. *Structure* **2017**, *25*, 1506-1518.e4, doi:10.1016/j.str.2017.07.019.

28. Azam, F.; Bello, M. Microsecond MD Simulations to Explore the Structural and Energetic Differences between the Human RXRα-PPARγ vs. RXRα-PPARγ-DNA. *Molecules* **2022**, *27*, 5778, doi:10.3390/molecules27185778.

29. Lemkul, J.A.; Lewis, S.N.; Bassaganya-Riera, J.; Bevan, D.R. Phosphorylation of PPARγ Affects the Collective Motions of the PPARγ-RXRα-DNA Complex. *PLoS ONE* **2015**, *10*, e0123984, doi:10.1371/journal.pone.0123984.

30. Zheng, J.; Corzo, C.; Chang, M.R.; Shang, J.; Lam, V.Q.; Brust, R.; Blayo, A.-L.; Bruning, J.B.; Kamenecka, T.M.; Kojetin, D.J.; et al. Chemical Crosslinking Mass Spectrometry Reveals the Conformational Landscape of the Activation Helix of PPARγ; a Model for Ligand-Dependent Antagonism. *Structure* **2018**, *26*, 1431-1439.e6, doi:10.1016/j.str.2018.07.007.

31. Frkic, R.L.; Pederick, J.L.; Horsfall, A.J.; Jovcevski, B.; Crame, E.E.; Kowalczyk, W.; Pukala, T.L.; Chang, M.R.; Zheng, J.; Blayo, A.-L.; et al. PPARγ Corepression Involves Alternate Ligand Conformation and Inflation of H12 Ensembles. *ACS Chem. Biol.* **2023**, *18*, 1115–1123, doi:10.1021/acschembio.2c00917.

32. Mottin, M.; Souza, P.C.T.; Skaf, M.S. Molecular Recognition of PPARγ by Kinase Cdk5/P25: Insights from a Combination of Protein–Protein Docking and Adaptive Biasing Force Simulations. *J. Phys. Chem. B* **2015**, *119*, 8330–8339, doi:10.1021/acs.jpcb.5b04269.

33. Montanari, R.; Capelli, D.; Yamamoto, K.; Awaishima, H.; Nishikata, K.; Barendregt, A.; Heck, A.J.R.; Loiodice, F.; Altieri, F.; Paiardini, A.; et al. Insights into PPARγ Phosphorylation and Its Inhibition Mechanism. *J. Med. Chem.* **2020**, *63*, 4811–4823, doi:10.1021/acs.jmedchem.0c00048.

34. Malospirito, C.C.; Jara, G.E.; Antunes, V.U.; Elias, G.B.; Dias, M.M.G.; Batista, F.A.H.; De Oliveira, P.S.L.; Figueira, A.C.M. The SIRT1 N-Terminal Domain as a Common Binding Interface for PPARΓ Anchoring. *Proteins* **2025**, *93*, 2167–2181, doi:10.1002/prot.70022.

35. Malospirito, C.C.; Dias, M.M.G.; Jara, G.E.; Avelino, T.M.; Elias, G.B.; De Oliveira, P.S.L.; Figueira, A.C.M. Interplay of Ser273 Phosphorylation and K268 and K293 Acetylation in PPARΓ : Implications for PPARΓ Activation. *Proteins* **2026**, prot.70140, doi:10.1002/prot.70140.

36. Biswas, A.; Saha, S.; Sarmadhikari, D.; Bisht, K.S.; Asthana, S.; Maiti, T.K. Bisphenol-A Mediated Ubiquitinome Alteration Triggers PPAR-Alpha Ubiquitination, Affecting Trophoblast Cell Migration 2026.

37. Agostini, M.; Schoenmakers, E.; Mitchell, C.; Szatmari, I.; Savage, D.; Smith, A.; Rajanayagam, O.; Semple, R.; Luan, J.; Bath, L.; et al. Non-DNA Binding, Dominant-Negative, Human PPARγ Mutations

Cause Lipodystrophic Insulin Resistance. *Cell Metabolism* **2006**, *4*, 303–311, doi:10.1016/j.cmet.2006.09.003.

38. Monajemi, H.; Zhang, L.; Li, G.; Jeninga, E.H.; Cao, H.; Maas, M.; Brouwer, C.B.; Kalkhoven, E.; Stroes, E.; Hegele, R.A.; et al. Familial Partial Lipodystrophy Phenotype Resulting from a Single-Base Mutation in Deoxyribonucleic Acid-Binding Domain of Peroxisome Proliferator-Activated Receptor-γ. *The Journal of Clinical Endocrinology & Metabolism* **2007**, *92*, 1606–1612, doi:10.1210/jc.2006-1807.

39. Lori, C.; Pasquo, A.; Montanari, R.; Capelli, D.; Consalvi, V.; Chiaraluce, R.; Cervoni, L.; Loiodice, F.; Laghezza, A.; Aschi, M.; et al. Structural Basis of the Transactivation Deficiency of the Human PPARγ F360L Mutant Associated with Familial Partial Lipodystrophy. *Acta Crystallogr D Biol Crystallogr* **2014**, *70*, 1965–1976, doi:10.1107/S1399004714009638.

40. Petrosino, M.; Lori, L.; Pasquo, A.; Lori, C.; Consalvi, V.; Minicozzi, V.; Morante, S.; Laghezza, A.; Giorgi, A.; Capelli, D.; et al. Single-Nucleotide Polymorphism of PPARγ, a Protein at the Crossroads of Physiological and Pathological Processes. *IJMS* **2017**, *18*, 361, doi:10.3390/ijms18020361.

41. Stalin, A.; Lin, D.; Josephine Princy, J.; Feng, Y.; Xiang, H.; Ignacimuthu, S.; Chen, Y. Computational Analysis of Single Nucleotide Polymorphisms (SNPs) in PPAR Gamma Associated with Obesity, Diabetes and Cancer. *Journal of Biomolecular Structure and Dynamics* **2022**, *40*, 1843–1857, doi:10.1080/07391102.2020.1835724.

42. Taghvaei, S.; Saremi, L. Molecular Dynamics Simulation and Essential Dynamics of Deleterious Proline 12 Alanine Single-Nucleotide Polymorphism in PPARγ2 Associated with Type 2 Diabetes, Cardiovascular Disease, and Nonalcoholic Fatty Liver Disease. *PPAR Research* **2022**, *2022*, 1–10, doi:10.1155/2022/3833668.

43. Madsen, M.S.; Broekema, M.F.; Madsen, M.R.; Koppen, A.; Borgman, A.; Gräwe, C.; Thomsen, E.G.K.; Westland, D.; Kranendonk, M.E.G.; Koerkamp, M.G.; et al. PPARγ Lipodystrophy Mutants Reveal Intermolecular Interactions Required for Enhancer Activation. *Nat Commun* **2022**, *13*, 7090, doi:10.1038/s41467-022-34766-9.

44. Ruscica, M.; Busnelli, M.; Runfola, E.; Corsini, A.; Sirtori, C.R. Impact of PPAR-Alpha Polymorphisms—The Case of Metabolic Disorders and Atherosclerosis. *IJMS* **2019**, *20*, 4378, doi:10.3390/ijms20184378.

45. Jeninga, E.H.; Van Beekum, O.; Van Dijk, A.D.J.; Hamers, N.; Hendriks-Stegeman, B.I.; Bonvin, A.M.J.J.; Berger, R.; Kalkhoven, E. Impaired Peroxisome Proliferator-Activated Receptor γ Function through Mutation of a Conserved Salt Bridge (R425C) in Familial Partial Lipodystrophy. *Molecular Endocrinology* **2007**, *21*, 1049–1065, doi:10.1210/me.2006-0485.

46. Genest, D.; Garnier, N.; Arrault, A.; Marot, C.; Morin-Allory, L.; Genest, M. Ligand-Escape Pathways from the Ligand-Binding Domain of PPARγ Receptor as Probed by Molecular Dynamics Simulations. *Eur Biophys J* **2008**, *37*, 369–379, doi:10.1007/s00249-007-0220-9.

47. Aci-Sèche, S.; Genest, M.; Garnier, N. Ligand Entry Pathways in the Ligand Binding Domain of PPARγ Receptor. *FEBS Letters* **2011**, *585*, 2599–2603, doi:10.1016/j.febslet.2011.07.014.

48. Liberato, M.V.; Nascimento, A.S.; Ayers, S.D.; Lin, J.Z.; Cvoro, A.; Silveira, R.L.; Martínez, L.; Souza, P.C.T.; Saidemberg, D.; Deng, T.; et al. Medium Chain Fatty Acids Are Selective Peroxisome

Proliferator Activated Receptor (PPAR) γ Activators and Pan-PPAR Partial Agonists. *PLoS ONE* **2012**, *7*, e36297, doi:10.1371/journal.pone.0036297.

49. Hughes, T.S.; Giri, P.K.; De Vera, I.M.S.; Marciano, D.P.; Kuruvilla, D.S.; Shin, Y.; Blayo, A.-L.; Kamenecka, T.M.; Burris, T.P.; Griffin, P.R.; et al. An Alternate Binding Site for PPARγ Ligands. *Nat Commun* **2014**, *5*, 3571, doi:10.1038/ncomms4571.

50. Kamata, S.; Oyama, T.; Saito, K.; Honda, A.; Yamamoto, Y.; Suda, K.; Ishikawa, R.; Itoh, T.; Watanabe, Y.; Shibata, T.; et al. PPARα Ligand-Binding Domain Structures with Endogenous Fatty Acids and Fibrates. *iScience* **2020**, *23*, 101727, doi:10.1016/j.isci.2020.101727.

51. Liu, X.; Jing, Z.; Jia, W.-Q.; Wang, S.-Q.; Ma, Y.; Xu, W.-R.; Liu, J.-W.; Cheng, X.-C. Identification of Novel PPARα/γ Dual Agonists by Virtual Screening, ADMET Prediction and Molecular Dynamics Simulations. *Journal of Biomolecular Structure and Dynamics* **2018**, *36*, 2988–3002, doi:10.1080/07391102.2017.1373706.

52. D'Aniello, E.; Fellous, T.; Iannotti, F.A.; Gentile, A.; Allarà, M.; Balestrieri, F.; Gray, R.; Amodeo, P.; Vitale, R.M.; Di Marzo, V. Identification and Characterization of Phytocannabinoids as Novel Dual PPARα/γ Agonists by a Computational and in Vitro Experimental Approach. *Biochimica et Biophysica Acta (BBA) - General Subjects* **2019**, *1863*, 586–597, doi:10.1016/j.bbagen.2019.01.002.

53. Iannotti, F.A.; De Maio, F.; Panza, E.; Appendino, G.; Taglialatela-Scafati, O.; De Petrocellis, L.; Amodeo, P.; Vitale, R.M. Identification and Characterization of Cannabimovone, a Cannabinoid from Cannabis Sativa, as a Novel PPARγ Agonist via a Combined Computational and Functional Study. *Molecules* **2020**, *25*, 1119, doi:10.3390/molecules25051119.

54. Kumar, A.P.; Mandal, S.; P, P.; Faizan, S.; Kumar, B.R.P.; Dhanabal, S.P.; Justin, A. Rational Design, Molecular Docking, Dynamic Simulation, Synthesis, PPAR-γ Competitive Binding and Transcription Analysis of Novel Glitazones. *Journal of Molecular Structure* **2022**, *1265*, 133354, doi:10.1016/j.molstruc.2022.133354.

55. Kumar, A.P.; P, P.; Mandal, S.; Prashantha Kumar, B.R.; Raju, R.M.; Dhanabal, S.P.; Rajagopal, K.; G, R.; X, P.N.; Justin, A. Computational Studies, Synthesis, in-Vitro Binding and Transcription Analysis of Novel Imidazolidine-2,4-dione and 2-Thioxo Thiazolidine-4-One Based Glitazones for Central PPAR-γ Agonism. *Journal of Molecular Structure* **2023**, *1285*, 135503, doi:10.1016/j.molstruc.2023.135503.

56. Haider, S.; Shafiq, M.; Siddiqui, A.R.; Sardar, M.; Mushtaq, M.; Shafeeq, S.; Nur-e-Alam, M.; Ahmad, A.; Ul-Haq, Z. Uncovering PPAR-γ Agonists: An Integrated Computational Approach Driven by Machine Learning. *Journal of Molecular Graphics and Modelling* **2024**, *129*, 108742, doi:10.1016/j.jmgm.2024.108742.

57. She, Y.; Zhao, J.; Ren, S.; Zhang, L.; Zhang, S.; Yang, Z. Molecular Insight into Pemetrexed as a Partial Agonist of PPAR$_{\gamma}$ through Molecular Dynamics Simulations. *Phys. Chem. Chem. Phys.* **2026**, *28*, 742–750, doi:10.1039/D5CP03840E.

58. Lokhande, K.B.; Ballav, S.; Yadav, R.S.; Swamy, K.V.; Basu, S. Probing Intermolecular Interactions and Binding Stability of Kaempferol, Quercetin and Resveratrol Derivatives with PPAR-γ: Docking, Molecular Dynamics and MM/GBSA Approach to Reveal Potent PPAR- γ Agonist against Cancer. *Journal of Biomolecular Structure and Dynamics* **2022**, *40*, 971–981, doi:10.1080/07391102.2020.1820380.

59. Prabitha, P.; Shanmugarajan, D.; Kumar, T.D.A.; Kumar, B.R.P. Multi-Conformational Frame from Molecular Dynamics as a Structure-Based Pharmacophore Model for Mapping, Screening and Identifying Ligands against PPAR-γ: A New Protocol to Develop Promising Candidates. *Journal of Biomolecular Structure and Dynamics* **2022**, *40*, 2663–2673, doi:10.1080/07391102.2020.1841677.

60. Perricone, U.; Wieder, M.; Seidel, T.; Langer, T.; Padova, A.; Almerico, A.M.; Tutone, M. A Molecular Dynamics–Shared Pharmacophore Approach to Boost Early-Enrichment Virtual Screening: A Case Study on Peroxisome Proliferator-Activated Receptor α. *ChemMedChem* **2017**, *12*, 1399–1407, doi:10.1002/cmdc.201600526.

61. Ahmed, S.; Islam, N.; Shahinozzaman, M.; Fakayode, S.O.; Afrin, N.; Halim, M.A. Virtual Screening, Molecular Dynamics, Density Functional Theory and Quantitative Structure Activity Relationship Studies to Design Peroxisome Proliferator-Activated Receptor-γ Agonists as Anti-Diabetic Drugs. *Journal of Biomolecular Structure and Dynamics* **2021**, *39*, 728–742, doi:10.1080/07391102.2020.1714482.

62. Mandal, S.K.; Muzaffar-Ur-Rehman, M.; Puri, S.; Kumar, B.K.; Sharma, P.K.; Sankaranarayanan, M.; Deepa, P.R. Unraveling PPARβ/δ Nuclear Receptor Agonists *via* a Drug-Repurposing Approach: HTVS-Based Ligand Identification, Molecular Dynamics, Pharmacokinetics, and *in Vitro* Anti-Steatotic Validation. *RSC Adv.* **2025**, *15*, 10622–10633, doi:10.1039/D4RA09055A.

63. Chen, K.-C.; Chang, S.-S.; Huang, H.-J.; Lin, T.-L.; Wu, Y.-J.; Chen, C.Y.-C. Three-in-One Agonists for PPAR-α, PPAR-γ, and PPAR-δ from Traditional Chinese Medicine. *Journal of Biomolecular Structure and Dynamics* **2012**, *30*, 662–683, doi:10.1080/07391102.2012.689699.

64. Mandal, S.K.; Puri, S.; Kumar, B.K.; Muzaffar-Ur-Rehman, M.; Sharma, P.K.; Sankaranarayanan, M.; Deepa, P.R. Targeting Lipid-Sensing Nuclear Receptors PPAR (α, γ, β/δ): HTVS and Molecular Docking/Dynamics Analysis of Pharmacological Ligands as Potential Pan-PPAR Agonists. *Mol Divers* **2024**, *28*, 1423–1438, doi:10.1007/s11030-023-10666-y.

65. Mandal, S.K.; Muzaffar-Ur-Rehman, M.; Puri, S.; Sharma, P.K.; Murugesan, S.; Deepa, P.R. In Silico and in Vitro Investigations Reveal Pan-PPAR Agonist Activity and Anti-NAFLD Efficacy of Polydatin by Modulating Hepatic Lipid-Energy Metabolism. *Sci Rep* **2025**, *15*, 26995, doi:10.1038/s41598-025-12357-0.

66. Feng, X.-Y.; Jia, W.-Q.; Liu, X.; Jing, Z.; Liu, Y.-Y.; Xu, W.-R.; Cheng, X.-C. Identification of Novel PPARα/γ Dual Agonists by Pharmacophore Screening, Docking Analysis, ADMET Prediction and Molecular Dynamics Simulations. *Computational Biology and Chemistry* **2019**, *78*, 178–189, doi:10.1016/j.compbiolchem.2018.11.023.

67. Nath, V.; Agrawal, R.; Kumar, V. Structure Based Docking and Molecular Dynamics Studies: Peroxisome Proliferator-Activated Receptors –α/γ Dual Agonists for Treatment of Metabolic Disorders. *Journal of Biomolecular Structure and Dynamics* **2020**, *38*, 511–523, doi:10.1080/07391102.2019.1581089.

68. Chen, K.-C.; Chen, C.Y.-C. *In Silico* Identification of Potent PPAR-γ Agonists from Traditional Chinese Medicine: A Bioactivity Prediction, Virtual Screening, and Molecular Dynamics Study. *Evidence-Based Complementary and Alternative Medicine* **2014**, *2014*, 192452, doi:10.1155/2014/192452.

69. Gangadhariah, M.; Pardhi, T.; Ravilla, J.; Chandra, S.; Singh, S.A. Citrus Nutraceutical Eriocitrin and Its Metabolites Are Partial Agonists of Peroxisome Proliferator-Activated Receptor Gamma (PPARγ): A Molecular Docking and Molecular Dynamics Study. *Journal of Biomolecular Structure and Dynamics* **2023**, *41*, 11373–11393, doi:10.1080/07391102.2022.2162127.

70. Wong, A.R.; Yang, A.W.H.; Gill, H.; Lenon, G.B.; Hung, A. Mechanisms of Nelumbinis Folium Targeting PPARγ for Weight Management: A Molecular Docking and Molecular Dynamics Simulations Study. *Computers in Biology and Medicine* **2023**, *166*, 107495, doi:10.1016/j.compbiomed.2023.107495.

71. Singh, N.; Pal, A.; Rana, D.; Sharma, P.; Thapliyal, A.; Mitra, D.; Sami, R.; Alsulaimani, F.; Basri, A.M.; Alnajeebi, A.M.; et al. *In Silico* Computational Screening and Molecular Dynamics Analysis Unveil *Moringa Oleifera* Phytochemicals as a Promising PPAR-γ Receptor Agonist for Targeted Diabetes Management Strategies. *Open Chemistry* **2026**, *24*, 20250216, doi:10.1515/chem-2025-0216.

72. Bhongade, B.A.; Ali, A.A.; Amnerkar, N.D.; Khadse, S.C.; Lokwani, D.K.; Patil, R.B.; Arooj, M.; Venkataraman, B.; Pessia, M.; Ojha, S.; et al. Natural Compounds as PPAR-γ Modulators in Inflammatory Bowel Disease: Insights from Molecular Docking and Dynamics Simulations. *Letters in Drug Design & Discovery* **2025**, *22*, 100345, doi:10.1016/j.lddd.2026.100345.

73. Zhao, C.; Liu, N.; Samad, A.; Tian, Z.; Xu, H.; Tian, J. Neferine Alleviated Corticosterone-Induced Depressive-like Behaviors in Mice via the Inhibition of TNF/NF-κB Pathway by Binding to PPARγ. *Mol Neurobiol* **2026**, *63*, 374, doi:10.1007/s12035-025-05659-x.

74. Ballav, S.; Lokhande, K.B.; Yadav, R.S.; Ghosh, P.; Swamy, K.V.; Basu, S. Exploring Binding Mode Assessment of Novel Kaempferol, Resveratrol, and Quercetin Derivatives with PPAR-α as Potent Drug Candidates against Cancer. *Mol Divers* **2023**, *27*, 2867–2885, doi:10.1007/s11030-022-10587-2.

75. Ayenero, M.E.; Oduola, T.M. An in Silico Studies of Solanum Erianthum Compounds as PPAR-α and TGF-βR1 Modulators in Oxidative Stress-Induced Liver Injury. *In Silico Research in Biomedicine* **2026**, *2*, 100197, doi:10.1016/j.insi.2026.100197.

76. Muralikumar, S.; Vetrivel, U.; Narayanasamy, A.; N. Das, U. Probing the Intermolecular Interactions of PPARγ-LBD with Polyunsaturated Fatty Acids and Their Anti-Inflammatory Metabolites to Infer Most Potential Binding Moieties. *Lipids Health Dis* **2017**, *16*, 17, doi:10.1186/s12944-016-0404-3.

77. Lian, Y.-E.; Wang, M.; Ma, L.; Yi, W.; Liao, S.; Gao, H.; Zhou, Z. Identification of Novel PPARγ Partial Agonists Based on Virtual Screening Strategy: In Silico and In Vitro Experimental Validation. *Molecules* **2024**, *29*, 4881, doi:10.3390/molecules29204881.

78. Kuo, K.-T.; Bdiri, B.; He, Y.; Garcia-Ordonez, R.D.; McDougal, D.P.; Ruiz, C.; Chang, M.R.; Cameron, M.D.; Bruning, J.B.; Kamenecka, T.M.; et al. Structural Determinants of Non-Covalent PPARγ Inverse Agonism and Their Therapeutic Implications. *Nat Commun* **2025**, *17*, 890, doi:10.1038/s41467-025-67608-5.

79. Padhy, I.; Banerjee, B.; Achary, P.G.R.; Gupta, P.P.; Sharma, T. Design, Synthesis, 2D-QSAR, Molecular Dynamic Simulation, and Biological Evaluation of Topiramate–Phenolic Acid Conjugates as PPARγ Inhibitors. *Futur J Pharm Sci* **2024**, *10*, 44, doi:10.1186/s43094-024-00617-1.

80. Wang, Z.; Shui, K.; Zhang, Z.; Chen, Y.; Yang, N.; Ji, S.; Shen, P.; Tian, Q. Discovery of a Novel Binding Pocket in PPARγ for Partial Agonists: Structure-Based Virtual Screening Identifies Ginsenoside

Rg5 as a Partial Agonist Promoting Beige Adipogenesis. *Front. Chem.* **2025**, *13*, 1579445, doi:10.3389/fchem.2025.1579445.

81. Liu, Y.; Liu, W.; Na, R.; Zhao, X. Discovery of PPARγ Partial Agonists for Treatment of Type 2 Diabetes Based on an Integrated Virtual Screening Strategy That Combines Fragment Molecular Orbital Calculations, Machine Learning, Molecular Docking, Interaction Fingerprint Filtering, and Molecular Dynamics Simulations. *J. Phys. Chem. B* **2026**, *130*, 961–979, doi:10.1021/acs.jpcb.5c06470.

82. Kaya, S.; Tatar-Yılmaz, G.; Aktar, B.S.K.; Emre, E.E.O. Discovery of New Dual-Target Agents Against PPAR-γ and α-Glucosidase Enzymes with Molecular Modeling Methods: Molecular Docking, Molecular Dynamic Simulations, and MM/PBSA Analysis. *Protein J* **2024**, *43*, 577–591, doi:10.1007/s10930-024-10196-y.

83. Kagimbo, N.M.; Mwankuna, C.J.; Mabiki, F.P. In Silico Evaluation of N-Methyl-1-Adamantaneacetamide and 3',4'-Di-O-Methyl Ellagic Acid-4α-La-Rhamnopyranoside from Asystasia Gangetica and Synadenium Glaucescens Pax as Potential Antidiabetic Agents. *Discov. Pharm. Sci.* **2026**, *2*, 3, doi:10.1007/s44395-025-00036-y.

84. Jain, S.; Vadia, N.; Gupta, S.; Asati, V.; Irani, M. Integrated Strategies for Designing Novel Thiazolidine-2, 4-Dione-Oxazole Derivatives as PPAR-γ Agonists: 3D-QSAR, Virtual Screening and Molecular Dynamics Simulations. *J. Comput. Biophys. Chem.* **2026**, *25*, 375–413, doi:10.1142/S2737416525500516.
